# Distributed Non-Invasive Electrical Monitoring and Short-Term Aggregate Load Forecasting for Wastewater Pumping Stations Using Custom Sensor Nodes and Neural Networks

**DOI:** 10.3390/s26206445

**Published:** 2026-10-12

**Authors:** Filippo Laganà

**Affiliations:** Laboratory of Biomedical Applications Technologies and Sensors (BATS), Department of Health Science, “Magna Græcia” University, 88100 Catanzaro, Italy; filippo.lagana@unicz.it

**Keywords:** monitoring, forecasting, wastewater, pumping, sensors

## Abstract

Continuous electrical data can support both monitoring and short-term forecasting in distributed infrastructures. This study presents an end-to-end framework for non-invasive current monitoring and forecasting of the platform-derived aggregate load of wastewater pumping stations. The deployed architecture combines SCT current sensors, Arduino Uno acquisition, a custom EasyEDA-designed electronic interface, Raspberry Pi-based storage and communication, and remote data aggregation. Fourteen stations comprising 23 three-phase pumps are covered by 69 sensing channels. The available dataset comprises 720 consecutive hourly observations. A rolling-origin protocol uses 18 days for initial fitting, five daily origins for hyperparameter selection, and seven held-out daily origins for testing, yielding 168 out-of-sample predictions. A seasonal-naïve benchmark was compared with a linear autoregressive model, a multilayer perceptron (MLP), and an RBF–GRNN. None of the three fitted models showed a statistically conclusive reduction in MAE relative to the seasonal-naïve benchmark, because all paired block-bootstrap 95% confidence intervals for the MAE differences included zero. Descriptively, among the four prespecified models, the MLP achieved the lowest MAE (0.441 kW) and MAPE (3.266%), whereas the RBF–GRNN achieved the lowest RMSE (0.525 kW) and the highest R^2^ (0.918). An exploratory post hoc LSTM analysis yielded an MAE of 0.424 kW but a higher RMSE (0.637 kW) and maximum error (2.959 kW); it likewise did not demonstrate a statistically conclusive advantage over either the MLP or the seasonal-naïve benchmark. The results demonstrate a modular sensing-to-forecasting workflow while showing that the observed differences among models require validation over longer monitoring periods.

## 1. Introduction

The progressive digitalisation of electrical and industrial infrastructures has transformed electrical monitoring from an occasional measurement activity into a continuous information process supporting energy assessment, operational supervision and demand forecasting. Non-invasive current sensors, microcontrollers, single-board computers, and Internet of Things (IoT) technologies now enable sensing, processing, storage, and communication functions to be integrated within modular electronic architectures. Low-cost wireless smart meters and IoT-based systems have demonstrated the feasibility of acquiring electrical quantities, remotely transmitting measurements and monitoring selected power-quality parameters [1,2,3]. Recent work has also investigated deep-learning approaches to modelling energy consumption in wastewater pumping systems [4]. More recent IoT-enabled meters have extended these capabilities through real-time communication, local processing and interoperable energy-management functions [5,6]. Data-driven monitoring and alarm functions have also been applied to pumping-station operating parameters [7], while open-source and device-level metering platforms have promoted system replicability and distributed installation [8,9]. Despite these developments, many available solutions remain primarily focused on measurement and visualisation and do not integrate a forecasting function directly connected to the acquired field data. The reduction of measurement invasiveness represents another important direction in electrical monitoring. Non-intrusive load-monitoring techniques can distinguish individual operating loads from aggregate electrical measurements, thereby reducing the number of sensors required within an installation [10]. Their principal advantage is the possibility of identifying load contributions without modifying the monitored equipment; however, their accuracy depends on the availability of representative training data and on the separability of electrical signatures. Smart-meter data have also been used for anomaly detection and power-quality assessment [11]. Distributed monitoring strategies have been extended to transmission-line parameters, microgrid loads, renewable energy communities and high-frequency energy-disaggregation systems [12,13,14,15]. Increasing the sampling frequency improves the observability of rapid electrical events, but also increases storage requirements, communication traffic, and computational cost. The temporal resolution should therefore be selected according to the monitoring objective and the operational dynamics of the observed infrastructure. Wastewater pumping stations constitute a relevant application for distributed electrical monitoring because their pumps are generally installed at geographically separated sites and operate intermittently according to inflow, storage level, and local control conditions. Their combined activation generates recurrent but variable aggregate-load profiles. Monitoring these profiles can improve operational awareness and provide the information required for short-term planning. Nevertheless, wastewater pumping applications introduce practical constraints that are not always considered in conventional forecasting studies, including the need for non-invasive installation, configurable acquisition channels, local data buffering, and remote communication from multiple stations. An effective solution must therefore consider the entire chain extending from current sensing to the construction and analysis of the aggregate electrical-load series. In parallel with the development of connected metering systems, the availability of electrical time series has encouraged the use of machine-learning methods for short-term load forecasting. Adaptive recurrent networks have been proposed to address variations in load behaviour and concept drift [16]. Comparative investigations of long short-term memory, gated recurrent unit and dropout-based recurrent architectures have confirmed their ability to represent nonlinear temporal dependencies [17,18,19]. Feature selection, genetic algorithms and metaheuristic optimisation procedures have subsequently been introduced to configure LSTM models and multisequence forecasting architectures [20,21]. These approaches can improve predictive performance and reduce dependence on manual parameter selection, but they expand the optimisation space and require sufficiently representative datasets. LSTM networks have been employed for other issues related to the energy sector apart from load forecasting, as these networks have the ability to maintain and update their internal states in an iterative way due to their recurrent nature and gating mechanism. The input gate, forget gate, and output gate control the amount of information that will be kept by the network from previous time steps; hence, the LSTM network is well suited to analyse electrical sequences with the properties of temporal dependencies, non-stationarity, and delay. With respect to renewable energy forecasting, one study combines signal processing and variational mode decomposition with LSTM for wind power forecasting [22]. Another study combines particle swarm optimisation, variational mode decomposition, and LSTM in the context of photovoltaic power forecasting [23]. In electrochemical energy storage, LSTM networks combined with transfer learning have been used to estimate the state of health of lithium-ion battery packs under limited training-data conditions [24]. More directly related to sensor-based electrical monitoring, the study integrates current sensors, Arduino and Raspberry Pi-based electronics, and remote data transmission with an LSTM model for hourly current absorption prediction and a U-Net model for classifying thermographic anomalies in an industrial plant [25]. This study addresses current absorption at the machine level and the evaluation of thermographic conditions, while the present work examines the aggregated load of geographically distributed wastewater pumping stations according to a rolling-origin forecasting protocol. In monitoring the condition of power electronic devices, the study applies a hybrid 1D CNN–LSTM architecture to voltage sequences for the detection and localisation of single and double open-circuit faults in a five-level cascaded H-bridge inverter [26]. In this architecture, the convolutional component extracts the local features of the signal, while the LSTM represents their temporal evolution. The referenced studies demonstrate the application of LSTM networks to the prediction of renewable energy generation, battery health estimation, industrial electrical monitoring, and fault diagnosis in power converters, as well as the prediction of conventional energy demand. Their relevance to the present work is methodological, as they demonstrate how gated recurrent models represent temporal relationships in sequential electrical data, although their measured variables, system boundaries, and forecasting objectives differ from those considered here. In the present study, the exploratory LSTM receives only the same preceding 24-hourly platform-derived aggregate-load values derived from the platform used by the MLP, without additional electrical, meteorological, or operational covariates. It also uses the same five validation origins, seven held-out test origins, and a 24-h forecast horizon. The comparison then examines the effect of modelling recurrent sequences under corresponding input and evaluation conditions. Since the LSTM was introduced after the inspection of the results of the four original models, its performance is interpreted descriptively as an exploratory comparison rather than a confirmatory demonstration of superiority over the feedforward MLP. Increasingly complex forecasting architectures have also been proposed. Recent system-level research has reviewed artificial-intelligence methods for maintenance and forecasting in interconnected power electronic systems [27], while attention mechanisms have been integrated into recurrent networks to identify informative temporal features [28]. Temporal convolutional networks have been combined with gradient-boosting algorithms, and bidirectional recurrent structures have been associated with deep-belief networks [29,30]. Hybrid convolutional–recurrent architectures can jointly extract local and temporal characteristics [31], whereas combinations of Random Forest and multilayer perceptron, as well as stacked generalisation models incorporating LightGBM, XGBoost and MLP predictors, attempt to reduce dependence on a single learning algorithm [32,33]. Deep networks have been applied to both short- and medium-term forecasting [34], while transformer models and ensemble procedures have been introduced to represent longer temporal relationships and combine heterogeneous predictors [35,36]. Although these architectures can achieve high forecasting accuracy, their increased number of parameters, training cost, and implementation complexity may be disproportionate when the available time series is limited or when the prediction function is intended for integration into a resource-constrained monitoring platform. The selection and representation of input variables are as important as the forecasting architecture. Permutation-importance methods and expert-guided Random Forest procedures can identify informative electrical, meteorological, and calendar variables while reducing input redundancy [37,38]. Conventional statistical models such as ARIMA remain useful forecasting references, whereas LSTM and gradient-boosting methods provide established alternatives for nonlinear sequence modelling [39,40,41]. Distributed datasets have motivated adaptive federated-learning strategies aimed at preserving data locality [42]. Comparative evaluations of LSTM and XGBoost models, studies involving local energy communities and graph-based recurrent networks further demonstrate that forecasting performance depends on the aggregation level, temporal resolution, and relationships among monitored nodes [43,44,45]. Graph-based architectures can explicitly represent spatial dependencies, but require prior knowledge of the connections among measurement points and may be unnecessarily complex when the forecasting target is a single aggregate series. Reviews of low-voltage load forecasting indicate that user heterogeneity, data availability and temporal resolution strongly affect model performance [46]. Other studies have investigated simulation-based energy optimisation for wastewater pumping systems [47], fuzzy clustering combined with deep learning [48], contextual variables derived from smart meters [49], and multifunctional Industrial IoT architectures [50]. These methods provide advanced analytical capabilities but also introduce additional processing stages and implementation requirements. Recent reviews confirm that no forecasting model is universally optimal and that the appropriate configuration depends on the forecast horizon, aggregation level, available variables, and computational constraints [51,52]. Hybrid one-step-ahead models, regularised neural networks and comparisons involving different network depths similarly show that greater structural complexity does not automatically guarantee better generalisation [53,54,55]. Equation-based modelling has also been applied in other engineering domains to analyse and forecast spatially distributed thermal-stress fields [56], providing a cross-domain example of a physically interpretable forecasting approach. The integration of artificial intelligence with distributed electrical-measurement systems is increasingly investigated [57], although reproducibility, validation design, data requirements and computational cost remain important concerns [58]. The metrological performance of current transformers is commonly characterised through ratio and phase errors determined by comparison with traceable reference instrumentation. Under non-sinusoidal operating conditions, magnetic-core nonlinearity and waveform distortion require additional assessment of harmonic measurement accuracy and measurement uncertainty [59]. Cross-domain studies of electronic interfaces, embedded acquisition, distributed sensing and data-driven signal interpretation provide broader design context for integrated sensor systems [60,61,62,63,64,65,66,67]. However, these studies do not provide evidence of the metrological performance of the SCT-based acquisition chain evaluated in the present work. Distorted current waveforms can further affect current-transformer accuracy [59,68]. These studies provide methodological context; they do not establish the accuracy of the current-to-load conversion used by the legacy platform. The contribution investigated here is a field-deployed sensing-to-forecasting workflow, whose quantitative forecasting results concern the archived platform output. The literature analysis reveals three principal gaps. First, numerous smart-metering systems provide acquisition, connectivity, and visualisation but do not incorporate a forecasting function directly connected to field measurements. Second, many load-forecasting studies employ public or externally generated datasets without describing the sensing and electronic architecture responsible for data generation. Third, comparisons among forecasting models frequently prioritise numerical accuracy without considering the consistency of the temporal inputs, realistic multi-step forecasting conditions, or the relationship between model complexity and the available amount of data. These limitations are particularly relevant for wastewater pumping infrastructure, where monitoring nodes must operate at distributed sites and the overall electrical load results from the combined activity of several intermittently activated pumps. The present study addresses these gaps through a distributed electrical-monitoring architecture deployed across 14 wastewater pumping stations comprising 23 three-phase pumps. Three non-invasive SCT current sensors were provided for each monitored pump, while custom electronic boards interfaced the sensing channels with Arduino Uno acquisition units. Raspberry Pi single-board computers managed local data storage and remote transmission to an online platform. The station-level contributions were processed to construct an aggregate time series consisting of 720 consecutive hourly load observations. The novelty of the study does not reside in introducing a new current sensor or a new neural-network architecture. Instead, it lies in the integration and experimental evaluation of the complete sensing-to-forecasting chain within an operational wastewater infrastructure. The principal contributions of this study are:

1. the design and field deployment of a modular, non-invasive electrical-monitoring architecture for geographically distributed wastewater pumping stations;
2. the integration of current sensing, embedded acquisition, local storage, cellular transmission, platform-level aggregation and short-term forecasting within a unified workflow;
3. the comparison of linear, multilayer and radial-basis forecasting models using a common chronological evaluation procedure and appropriate reference baselines;
4. the assessment of 24-h forecasting performance using the surviving hourly export, with the processed dataset and reported forecasts provided to support inspection of the analysis results.

The remainder of the manuscript is organised as follows. Section 2 describes the monitoring architecture, field configuration, data-processing procedure, forecasting models and evaluation metrics. Section 3 presents the hardware implementation, dataset assessment and forecasting results. Section 4 discusses the implications of the findings and compares the evaluated approaches with related studies. Section 5 summarises the principal conclusions.

## 2. Materials and Methods

The methodology establishes a traceable connection between the distributed sensing architecture, the construction of the aggregate electrical-load series and its subsequent use for short-term forecasting. The monitored infrastructure comprises 14 geographically distributed wastewater pumping stations containing a total of 23 three-phase pumps, with between one and three pumps installed at each station. Current-related observations were acquired locally at a nominal interval of one minute and transmitted to the remote platform, where platform-derived pump- and station-level load estimates were generated and subsequently combined. The available dataset consisted of 720 chronologically ordered hourly observations representing 30 complete days of aggregate operation. The methodology is organised into five subsections. Section 2.1 describes the conceptual design and functional architecture of the custom monitoring system. Section 2.2 defines the station configuration, sensor arrangement and measurement strategy. Section 2.3 formalises the acquisition, aggregation and pre-processing procedures used to construct the hourly time series. Section 2.4 presents the forecasting models, temporal-input representations and model-selection procedure. Finally, Section 2.5 defines the chronological evaluation protocol, reference baselines and error metrics. The corresponding hardware implementation, dataset verification, and forecasting outcomes are presented in Section 3, thereby ensuring direct correspondence between each methodological stage and its experimental assessment.

### 2.1. Design and Architecture of the Monitoring System

The monitoring system was conceived according to a modular and distributed architecture suitable for geographically separated wastewater pumping stations. The principal design requirements were the non-invasive acquisition of pump currents, scalability of the measurement channels, separation between signal acquisition and data management, local storage, and remote communication. Similar embedded monitoring systems demonstrate that multi-channel acquisition, modular sensor nodes, and IoT connectivity can facilitate the adaptation of the same architecture to installations with different electrical and operational configurations [69,70,71,72]. The proposed architecture comprises four functional levels: current sensing, signal interfacing and acquisition, local data management, and communication with the remote platform. The sensing level is based on non-invasive SCT current sensors connected to the conductors supplying the monitored pumps. Their signals are routed to dedicated inputs on a custom electronic interface board. The acquisition level is based on an Arduino Uno development board incorporating an ATmega328P microcontroller. The acquired samples are transferred to a Raspberry Pi single-board computer, which performs local data management and handles communication with the remote platform. Figure 1 connects the three organisational levels of the proposed framework. Panel A defines the distributed field deployment and its overall sensing capacity. Panel B expands a typical station node and shows how each three-phase pump contributes three SCT current-sensing channels to a scalable architecture of signal conditioning, data acquisition, and local data management. Panel C tracks the station records transferred remotely through platform-level aggregation, the construction of archived hourly series, the chronological rolling-origin evaluation, and the generation of 24-h forecasts.

The separation between the acquisition and data-management levels was introduced to improve modularity. The Arduino Uno is responsible for reading the signals provided by the custom interface board, whereas the Raspberry Pi manages local storage and transmission. Communication between the digital units is implemented through a USB connection. This configuration allows the acquisition procedure to remain unchanged if the communication technology or data-management software is subsequently modified. It also permits the number of sensing channels to be adapted to the pumps installed at each station without redesigning the complete monitoring architecture. The Raspberry Pi was selected as the local computing and communication unit because it provides USB connectivity, local memory, and support for external communication devices. The architecture was designed to store the acquired observations locally before transmission, thereby reducing dependence on continuous network availability. When remote connectivity is available, the stored records are transferred to the online platform, where the measurements from the individual stations can be organised, aggregated and prepared for subsequent analysis. The system therefore separates the time-critical acquisition process from remote storage and higher-level processing. The custom electronic interface was designed using EasyEDA Pro (version 2.2) and provides dedicated input and signal-conditioning paths for the SCT current sensors. The basic interface accommodates the three sensing channels associated with the phases of a monitored pump and conveys the conditioned signals to the Arduino Uno acquisition stage. At stations containing more than one pump, the sensing architecture was expanded by repeating the corresponding three-phase input groups. The Arduino Uno and Raspberry Pi perform separate functions: the former manages signal acquisition, whereas the latter performs data buffering, local storage, and remote transmission. The two digital units communicate through a USB connection. Figure 2a provides a functional representation of the connections among the SCT inputs, signal-conditioning stage, and Arduino Uno acquisition unit, whereas Figure 2b documents the available PCB layout. Figure 2a should not be interpreted as a complete electrical schematic because the surviving design documentation does not preserve the component values, detailed analogue topology, or ADC configuration required to reconstruct and independently validate the acquisition chain.

Figure 2a shows the functional arrangement of one three-phase sensing group, and Figure 2b shows the PCB layout. The installed sensors are SCT-013-000 split-core AC current transformers, with one sensor on each phase conductor of the 23 pumps, for a total of 69 sensors. The manufacturer’s specifications give a nominal primary-current range of 0–100 A and a secondary-current output of 0–50 mA at rated input, equivalent to a nominal ratio of 0.5 mA/A. The specified sensor accuracy is ±1%, and its linearity is ±3%. These are component specifications, not experimentally verified accuracy or precision values for the installed acquisition channels. The available documentation does not establish the burden-resistor values, complete analogue conditioning parameters, ADC settings, channel-specific calibration results, or response time of the complete measurement chain. The nominal one-minute reporting interval must not be interpreted as the sensor response time.

The nominal transformation ratio of 0.5 mA/A is reported here as the manufacturer-specified current-transfer sensitivity of the SCT-013-000 sensor. The ±1% accuracy and ±3% linearity values likewise refer only to the sensor specification and not to the precision of the complete sensing, conditioning and digitisation chain. No experimentally determined channel-level precision, repeatability or response time is available. The deployed configuration used only current transformers; no voltage, power-factor, wet-well-level, flow, rainfall or pump-state sensors were integrated into the surviving monitoring dataset. Consequently, the available information is insufficient to assign a traceable uncertainty budget to the installed measurement channels or to verify their accuracy, precision, repeatability and dynamic response under actual pump operating conditions.

The design architecture described allows each monitoring unit to be configured according to the number of pumps in the station, whilst keeping the measurement principle and the data management chain unchanged. The modular nature of the functional blocks also makes it possible to replace or upgrade individual components without having to redesign the entire system. The physical implementation of the monitoring assembly and its installation in an electrical cabinet are documented in Section 3.

### 2.2. Station Configuration and Measurement Strategy

The monitoring architecture was designed for wastewater pumping infrastructure in which electrical loads are geographically distributed and activated according to local operating requirements. Data-driven energy prediction, pumping-station parameter forecasting, and energy auditing have demonstrated the importance of combining continuous monitoring with analysis tools capable of supporting operational and energy-efficiency assessments [73,74,75]. Such applications benefit from multi-source data availability, but their implementation requires a scalable measurement strategy capable of accommodating stations with different numbers of pumps. The monitored infrastructure comprised 14 wastewater pumping stations and 23 three-phase pumps, with between one and three pumps installed at each station. The local monitoring configuration was adapted to the number of pumps present at the corresponding site. Three non-invasive SCT current sensors were assigned to each pump, with one sensor associated with each phase conductor. The complete distributed installation therefore comprised 69 current-sensing channels. Stations containing two or three pumps used multiple three-phase sensing groups connected to the local acquisition architecture. The clamp-type configuration enabled installation without cutting the pump conductors or introducing series measurement components, thereby reducing installation invasiveness and facilitating adaptation to existing electrical cabinets. However, current sensing alone does not provide a direct metrological measurement of active power unless voltage, phase displacement, and power factor are also known. Accordingly, the quantities used in the forecasting analysis are defined as platform-derived load estimates rather than direct measurements of active power. Active power requires simultaneous voltage and current information. For three phases, it is obtained from the time average of the phase-wise products of voltage and current; under balanced sinusoidal conditions, it can be expressed as $P=\sqrt{3}V_{LL}I_{L}\cos\phi .$ Neither voltage waveforms nor a time-varying power factor were recorded in the surviving dataset. Changes in motor operating conditions and power factor can therefore introduce an unknown, potentially nonlinear difference between the exported load estimate and true active power. The present data do not permit that difference to be quantified or corrected. The local acquisition unit collected one current value from each configured sensing channel at a nominal interval of one minute. The 69 installed devices are SCT-013-000 split-core AC current transformers, with three channels associated with each three-phase pump. Their secondary signals passed through the analogue interface before acquisition by the Arduino Uno. The surviving export does not establish whether each one-minute value was an instantaneous sample, an RMS estimate, or the result of another processing operation. The 30-day duration describes the available hourly aggregate series; individual channel sample counts, uptime, and continuous operation over the entire period cannot be verified because the per-channel records are unavailable. At the transducer output, the physical signal was an AC secondary current nominally proportional to the corresponding phase current. After passage through the analogue interface, the Arduino-based acquisition unit recorded one current-derived value per configured channel at a nominal interval of one minute. However, the surviving documentation does not establish whether this value represented an instantaneous sample, an RMS estimate, an average, a peak value, or another processed quantity. Therefore, neither the exact number of observations nor the effective acquisition duration can be reconstructed separately for each of the 69 channels. Acquisition was scheduled periodically, with local storage and subsequent transfer to the remote platform when connectivity was available. No traceable channel-by-channel calibration or validation against a reference ammeter, power analyser or calibrated current source is available. The verification performed in the present study is restricted to the surviving 720-point hourly aggregate export and includes checks of completeness, chronological ordering, duplicated timestamps, missing observations and invalid load values. These data-quality checks do not validate the original sensors, analogue front end, current-to-load conversion or hourly aggregation procedure. The system is therefore described as a distributed monitoring and remote-data platform, without claiming SCADA operation or verified real-time performance. The measurement interval was selected as a compromise between the representation of pump activation patterns and the amount of data requiring local storage and remote transmission. A shorter interval would provide more detailed information about transient events but would increase communication traffic and storage requirements. Conversely, a longer interval could reduce the ability to distinguish short operating cycles. The one-minute interval was therefore adopted for periodic monitoring of pump operation rather than for high-frequency analysis of electrical transients. The current values associated with each pump were transmitted to the remote platform through the local Raspberry Pi gateway. At platform level, the acquired quantities were processed to obtain the corresponding pump-load estimates. The contributions of the pumps installed at the same site were combined to determine the station-level load, while the loads associated with the 14 stations were subsequently summed to construct the aggregate electrical-load series. The forecasting target therefore represents the overall demand of the monitored infrastructure rather than the operation of an individual pump or station. The dataset available for the present analysis contains the platform-generated aggregate-load values rather than the original phase-current waveforms. Consequently, the study does not retrospectively reconstruct RMS current, voltage, or power factor from raw electrical signals. The exported values were stored in watts by the original platform and were converted into kilowatts during pre-processing. Because the underlying current-to-load conversion function and its calibration are no longer traceably documented, the kilowatt unit is retained solely as the nominal unit assigned by the legacy platform. It must not be interpreted as evidence of a calibrated or SI-traceable active-power measurement. This distinction preserves the traceability of the available dataset and avoids interpreting the exported load estimates as direct power-analyser measurements. The mathematical formulation of the pump-, station- and infrastructure-level aggregation is provided in Section 2.3. The main characteristics of the measurement configuration are summarised in Table 1.

The adopted configuration maintains the association among individual sensing channels, pumps and stations before calculating the aggregate infrastructure load. This hierarchical organisation enables local measurements to be traced to their respective operating units while providing a single chronological target for short-term forecasting.

Section 3 will document the physical integration of the sensors and electronic units within the electrical cabinet, whereas the following subsection formalises the processing applied to construct the 720-point hourly series.

### 2.3. Data Acquisition and Pre-Processing

At each pumping station, the local acquisition unit collects the current values associated with the three phases of each monitored pump. Let *I_s,p,l_*(*m*) denote the current value acquired at minute (*m*) from phase *l* of pump *p* installed at station *s*, where *l* ∈ {1,2,3}. The original remote platform converted the three-phase current values into a pump-load estimate through a processing function whose implementation and calibration parameters are no longer available for verification. The platform-derived pump-load estimate can therefore be formally represented by (1):

(1) $$P_{s,p}^{plat}\left(m\right)=G_{s,p}\left[I_{s,p,1}\left(m\right),I_{s,p,2}\left(m\right),I_{s,p,3}\left(m\right)\right]$$

where *G_s,p_*(*∙*) denotes the legacy current-to-load conversion function; its implementation and calibration parameters are unavailable. Equation (1) is therefore a formal representation of the unavailable legacy conversion procedure and not a validated physical model relating phase current to active power. Since the available dataset contains the exported load values rather than the raw current waveforms, the present analysis does not retrospectively estimate RMS current, phase displacement, or power factor. Accordingly, $P_{s,p}^{plat}\left(m\right)$ is treated as a platform-derived electrical-load estimate. Let *N*_s_ denote the number of pumps installed at station *s*. The total load associated with the station at minute *m* is defined in (2)

(2) $$P_{s}\left(m\right)=\sum_{p=1}^{N_{s}}P_{s,p}^{plat}\left(m\right),\;N_{s}\in \left\{1,2,3\right\}$$

The overall load of the monitored infrastructure is obtained by combining the contributions from the 14 wastewater pumping stations. The aggregate value is expressed in (3)

(3) $$P_{tot}\left(m\right)=\sum_{s=1}^{14}P_{s}\left(m\right)=\sum_{s=1}^{14}\sum_{p=1}^{N_{s}}P_{s,p}^{plat}(m)$$

The hierarchical formulation in (1)–(3) preserves the association among sensing channel, pump and station before the infrastructure-level load is calculated. The objective of the forecasting procedure is therefore to estimate the aggregate electrical demand represented by *P_tot_*(*m*), rather than the behaviour of an individual pump. The original platform generated one representative hourly aggregate-load value from the minute-level station records. Let $H_{n}$ denote the set of minute-level observations associated with hour *n*, and let A*_h_* denote the unknown hourly aggregation operator implemented by the legacy platform. The archived hourly value can therefore be represented in (4)

(4) $$P_{h}\left(n\right)=A_{h}\left(\left\{P_{tot}\left(m\right):m\in H_{n}\right\}\right)$$

The surviving dataset contains the hourly values produced by this procedure, whereas the original minute-level records and complete platform software are no longer available. Consequently, the exact aggregation operator in (4) cannot be independently re-executed or verified. The forecasting analysis therefore begins from the 720 chronologically ordered hourly values contained in the available export. Only if an hourly value is verified to represent the mean of calibrated active power can the corresponding electrical energy be obtained by multiplication by the one-hour interval, as shown in (5). This condition cannot be verified for the surviving export; Equation (5) is included solely to distinguish the units and is not used to derive an energy result in this study

(5) $$E_{h}\left(n\right)=P_{h}\left(n\right)∆t,\;∆t=1\;h$$

where *E_h_*(*n*) is expressed in kWh whilst *P_h_*(*n*) is expressed in kW. The forecasting models use the hourly load rather than the energy quantity defined in (5). The file available for the present study contains the results of the original hourly aggregation and does not include the minute-level phase-current observations. The exported load values were stored in watts. They were converted into kilowatts using (6)

(6) $$P_{kW}\left(n\right)=\frac{P_{raw}(n)}{1000}$$

where *P_raw_*(*n*) represents the stored value and *P*_kW_(*n*) the corresponding load expressed in kilowatts. The transformation in (6) changes only the unit of measurement and does not alter the temporal profile or relative variability of the series. The available sequence contains 30 complete days and 24 observations for each day. Let *q* ∈ {1, …, 30} denote the sequential day and *h* ∈ {1, …, 24} the hour within that day. The chronological index is constructed according to (7)

(7) $$n=24\left(q-1\right)+h,\;n=\left\{1,\;\ldots ,\;720\right\}$$

The relationship in (7) maintains chronological continuity between the twenty-fourth observation of one day and the first observation of the following day. Each sample is also associated with a day-of-week index *d*(*n*) ∈ {1, …, 7} and an hour-of-day index *h*(*n*) ∈ {1, …, 24}. Calendar variables are widely used to represent daily and weekly periodicity in short-term electrical-load forecasting [76,77]. Their cyclic transformation and use within the forecasting models are described in Section 2.4. Before model development, the exported dataset was checked for completeness, chronological ordering, duplicate temporal positions, and non-physical values. The sample mean and sample standard deviation of the aggregate load are calculated using (8):

(8) $$\bar{P}=\frac{1}{N}\sum_{n=1}^{N}P_{kW\left(n\right),\;}s_{p}=\sqrt{\frac{1}{N-1}\sum_{n=1}^{N}{\left[P_{kW}\left(n\right)-\bar{P}\right]}^{2}}$$

where *N* = 720. The relative variability of the series is quantified through the coefficient of variation defined in (9):

(9) $${CV}_{p}=\frac{s_{P}}{\bar{P}}\times 100$$

The completeness of the hourly dataset is assessed using the missing-data rate in (10)

(10) $$r_{miss}=\frac{N_{miss}}{N_{exp}}\times 100,\;N_{exp}=30\times 24=720$$

where *N_miss_* is the number of missing observations, and *N_exp_* is the expected number of samples. The verification procedure also checks for negative or zero values, duplicated chronological positions, incomplete daily sequences, and inconsistencies in the day-of-week progression. No automatic interpolation or replacement of extreme observations is performed. Load peaks and low-load intervals may correspond to actual combinations of pump activation and should not be removed solely on the basis of their statistical distance from the mean. An observation may be excluded only when there is explicit evidence of an acquisition or export error. The complete acquisition and pre-processing workflow is illustrated in Figure 3.

Figure 3 represents the sequence from the three-phase pump-current measurements to the platform-derived pump loads, station-level aggregation, combination of the 14 station loads, hourly aggregation by the legacy platform, whose exact operator is unavailable, unit conversion, data-quality assessment, and preparation of the chronological forecasting dataset. The distinction between operations performed by the original acquisition platform and procedures applied to the exported hourly file should remain visually evident. The resulting dataset structure is summarised in Table 2.

The processing stages defined in this subsection establish the correspondence between the distributed measurement architecture and the dataset used for forecasting. The adoption of an hourly temporal resolution represents a deliberate aggregation choice intended to generate a common infrastructure-level forecasting variable from measurements acquired at a finer temporal scale. This operation facilitates comparison across stations and reduces the dimensionality of the forecasting problem, but it may also attenuate short-duration transients associated with individual pump switching events. The present analysis therefore addresses short-term prediction of the hourly aggregate-load profile rather than the reconstruction or forecasting of high-frequency electrical dynamics. The influence of alternative aggregation intervals should be investigated in future studies to determine whether shorter temporal resolutions provide additional predictive information. Section 3 will consequently report the complete 720-point chronological profile, its empirical distribution, the descriptive statistics defined in (8) and (9), and the quality indicators introduced in (10). This correspondence ensures that each data-processing operation described in the methodology is supported by quantitative or graphical evidence in the Results. The electrical quantity used throughout the forecasting analysis corresponds to the aggregate load variable generated by the monitoring platform and exported in the available database. Accordingly, the values expressed in kW should not be interpreted as independently reconstructed active-power measurements obtained from simultaneous voltage and current waveform acquisition and power-factor estimation. Instead, they represent the electrical-load indicator produced by the platform processing chain for the monitored pumping infrastructure. The forecasting task investigated in this study is therefore specifically defined as the prediction of this platform-derived aggregate-load variable. This distinction is important when interpreting the reported forecasting errors, since the present analysis evaluates the ability of the models to reproduce the temporal evolution of the measured database variable rather than the absolute metrological accuracy of a power analyser.

### 2.4. Forecasting Models and Hyperparameter Selection

The forecasting analysis was designed to compare models characterised by different levels of complexity while maintaining a common temporal evaluation procedure. Four configurations were considered: a seasonal-naïve benchmark, a linear autoregressive model, a multilayer perceptron trained through backpropagation, and a radial basis function–generalised regression neural network (RBF–GRNN). The seasonal benchmark provides a reference based exclusively on daily periodicity, whereas the remaining models represent linear dependence, nonlinear dependence on lagged observations, and nonlinear regression based on calendar variables, respectively. The purpose of the model comparison was not to establish an exhaustive benchmark across all available time-series and machine-learning algorithms. Instead, four configurations were selected to represent progressively different forecasting principles within the limited size of the available dataset: repetition of the previous daily profile, linear dependence on historical observations, nonlinear feedforward learning from lagged load values, and nonlinear regression based on cyclic calendar information. The original comparison comprises four models and excludes recurrent, attention-based, and ensemble architectures. Section 2.4.7 introduces an LSTM as an exploratory comparator added during manuscript revision. A robust comparison involving a broader range of high-capacity architectures requires longer monitoring and more independent forecast origins. Feedforward neural networks provide relatively compact alternatives to deeper recurrent architectures [78,79], while GRNN models offer a non-iterative formulation based on Gaussian responses centred on the available samples [80]. All models were evaluated through 24-h forecasts. The linear and MLP models generated the 24 predicted values recursively, without accessing measured observations belonging to the forecast day. The seasonal-naïve and RBF–GRNN configurations produced the complete 24-h profile directly using, respectively, the previous-day load profile and calendar information available at the forecast origin. The comparison should therefore be interpreted as an evaluation of complete forecasting configurations rather than as an isolated comparison of neural-network architectures. In particular, the models differ not only in their mathematical formulation but also in the information used as input and in the strategy adopted to generate the 24-h forecast horizon.

The linear and MLP configurations exploit historical load observations and generate multi-step forecasts recursively, whereas the RBF–GRNN relies on calendar variables and directly estimates the complete daily profile. Consequently, the observed performance differences reflect the combined effects of model structure, input representation and forecasting mode.

#### 2.4.1. Rolling-Origin Organisation and Standardisation

The 720 observations were divided into an initial training interval, a multi-window validation interval and a multi-window test interval. The first 18 days, corresponding to 432 hourly observations, formed the initial training set. The subsequent five days were used for hyperparameter selection, while the final seven days were reserved for out-of-sample testing. Each validation or test fold comprised a complete 24-h forecasting horizon. For a generic forecast origin o, the fitting and evaluation sets are defined in (11):

(11) $$D_{fit}^{\left(o\right)}={\left\{P_{kW}(n)\right\}}_{n-1}^{o-1},\;D_{eval}^{\left(o\right)}={\left\{P_{kW}(n)\right\}}_{n=0}^{o+23}$$

The forecast origins used for validation and testing are reported in (12)

(12) $$O_{val}=\left\{433,457,481,505,529\right\},\;O_{test}=\left\{553,577,601,625,649,673,697\right\}$$

At each origin, the fitting interval was expanded to include all observations available before that forecast day. During validation, the resulting five 24-h forecasts were used exclusively for hyperparameter selection. Once the configuration of each model had been selected, its hyperparameters were fixed and were not modified using the seven test days. For each test fold, the model was refitted using only the observations preceding the corresponding forecast origin. This rolling-origin design reflects the operational condition in which a new daily forecast is generated after the measurements from the preceding days have become available [77]. The load values used by the linear and MLP models were standardised separately at each forecast origin. Let *μ_o_* and *s_o_* denote the mean and sample standard deviation calculated using $D_{fit}^{\left(o\right)}$. The standardised load is defined in (13)

(13) $${\tilde{P}}^{o}\left(n\right)=\frac{P_{kW}\left(n\right)-\mu_{o}}{s_{o}},\;n<o$$

The same *μ_o_* and *s_o_* values were used to transform the model outputs back into kilowatts. Recalculating the standardisation parameters at each origin prevents observations belonging to the forecast interval from influencing model fitting.

#### 2.4.2. Seasonal-Naïve Benchmark

The seasonal-naïve model assumes that the load profile of the forecast day is equal to that observed during the preceding day. For forecast origin, its prediction is defined in (14)

(14) $${\hat{P}}_{SN}^{o}\left(o+k\right)=P_{kW}\left(o+k-24\right),\;k=0,\;\ldots ,\;23$$

This benchmark does not require parameter estimation or iterative training. Its inclusion makes it possible to determine whether the more complex forecasting configurations provide an improvement over the daily periodicity already present in the measured series. The seasonal-naïve model is particularly relevant because the dataset exhibits recurrent 24-h operating patterns.

#### 2.4.3. Linear Autoregressive Model

The linear autoregressive model uses the most recent standardised load observations. At forecast origin, the input vector associated with time is defined in (15)

(15) $$x_{d}^{\left(o\right)}\left(n\right)={\left[{\tilde{P}}^{\left(o\right)}\left(n-1\right)\;{\tilde{P}}^{\left(o\right)}\left(n-2\right)\;{\tilde{P}}^{\left(o\right)}\left(n-d\right)\right]}^{T}.$$

The corresponding one-step model is expressed in (16)

(16) $${\hat{\tilde{P}}.}_{AR}^{\left(o\right)}\left(n\right)=b_{0}+\sum_{j=1}^{d}w_{j}{\tilde{P}}^{\left(o\right)}\left(n-j\right),$$

where *w_j_* denotes the coefficient associated with lag *j* and *b_0_* is the intercept. The coefficients were estimated using ordinary least squares. To ensure comparability with the MLP, both models were evaluated using the same candidate delays *d* ∈ {1,3,6,12,24,48,72,96}. For each candidate, the model was refitted at the five validation origins and used to generate a recursive 24-h forecast. Selection according to the mean validation MAE identified *d* = 96. During recursive forecasting, each predicted standardised value was appended to the input history and used to estimate the subsequent hour.

#### 2.4.4. Multilayer Perceptron with Backpropagation

The nonlinear autoregressive configuration consists of a feedforward multilayer perceptron with a single hidden layer and a linear output neuron. The input is the delayed vector defined in (15). The activation of hidden neuron *k* is calculated using (17):

(17) $$a_{k}\left(n\right)=tanh\left[b_{k}^{\left(1\right)}+\sum_{j=1}^{d}w_{kj}^{\left(1\right)}x_{d,j}\left(n\right)\right],\;k=1,\ldots ,\;H$$

where *H* is the number of hidden neurons. The network output is given by (18)

(18) $${\hat{\tilde{P}}}_{MLP}^{\left(o\right)}\left(n\right)=b^{\left(2\right)}+\sum_{k=1}^{H}w_{k}^{\left(2\right)}a_{k}(n)$$

The weights and biases were estimated by minimising the regularised loss function defined in (19)

(19) $$L_{MLP}=\frac{1}{N_{p}}\sum_{i=1}^{N_{p}}{\left[{\tilde{P}}_{i}-{\hat{\tilde{P}}}_{i}\right]}^{2}+\frac{\lambda }{2N_{p}}\left({\left\|W^{\left(1\right)}\right\|}_{F}^{2}+{\left\|W^{\left(2\right)}\right\|}_{2}^{2}\right),$$

where *N_p_* is the number of available training patterns and λ is the *L*_2_ regularisation coefficient. The same delay candidates used for the linear model were considered. Hidden-layer sizes *H* ∈ {8,16,32} were evaluated. All configurations used hyperbolic-tangent activation, an *L*_2_ coefficient of 10^−4^, the limited-memory Broyden–Fletcher–Goldfarb–Shanno optimisation algorithm, a maximum of 3000 iterations and a fixed pseudo-random seed of 42. The mean error over the five validation days selected *d* = 24 and *H* = 16. The 24-h forecasts were subsequently generated recursively using the same procedure adopted for the linear model.

#### 2.4.5. Cyclic RBF–GRNN Model

The fourth configuration is a Generalised Regression Neural Network based on radial Gaussian functions. Unlike the autoregressive models, the GRNN uses calendar variables available before the forecast interval. The hour of the day and day of the week were represented through sine and cosine components to preserve their cyclic nature. The input vector is defined in (20)

(20) $$x_{cal}(n)=\left[\begin{array}{c} \begin{array}{c} \sin\left\{2\pi \frac{h\left(n\right)-1}{24}\right\}\\ \cos\left\{2\pi \frac{h\left(n\right)-1}{24}\right\} \end{array}\\ \sin\left\{2\pi \frac{d\left(n\right)-1}{7}\right\}\\ \cos\left\{2\pi \frac{d\left(n\right)-1}{7}\right\} \end{array}\right]$$

This representation ensures that hours 24 and 1, as well as days 7 and 1, remain close in the input space. It also prevents the numerical scale of the hour variable from dominating the day-of-week variable. For each centre *c_j_*, corresponding to a sample in the fitting set, the radial response is defined in (21):

(21) $$\phi_{j}\left(x\right)=\exp\left[-\frac{{\left\|x-c_{j}\right\|}_{2}^{2}}{2\sigma^{2}}\right]$$

where σ represents the spread parameter. The GRNN prediction is obtained using the normalised weighted average in (22)

(22) $${\hat{\tilde{P}}}_{GRNN}\left(n\right)=\frac{\sum_{j=1}^{N_{fit}}{\tilde{P}}_{j}\phi_{j}(x)}{\sum_{j=1}^{N_{fit}}\phi_{j}(x)}$$

Smaller values of σ produce more localised responses, whereas larger values increase the smoothing applied to the calendar-dependent load profile. Candidate values from 0.05 to 1.50 were evaluated using an increment of 0.05. Selection over the five validation days identified σ = 0.30. The selected value lies within the explored interval rather than at one of its boundaries, indicating that the search range adequately encompassed the local optimum.

#### 2.4.6. Hyperparameter Selection and Final Forecasting Procedure

For model *r*, the optimal hyperparameter configuration $\theta_{r}^{*}$ was selected using (23):

(23) $$\theta_{r}^{*}=\underset{\theta \in \Theta }{\arg\min}\frac{1}{\left|O_{val}\right|}\sum_{o\in O_{val}}{MAE}_{24}^{\left(o\right)}\left(\theta \right),$$

where $\Theta_{r}$ is the candidate set for model *r*, and ${MAE}_{24}^{\left(o\right)}$ is the mean absolute error calculated over the 24-h forecast generated at origin *o*. Hyperparameter selection therefore depends on the average performance over five complete daily profiles rather than on one-step-ahead predictions. 

The rolling-origin procedure used for model selection and seven-day out-of-sample evaluation is illustrated in Figure 4.

The 720-h series was divided into an initial 18-day training interval, followed by five validation days used for hyperparameter selection and seven held-out test days. At each forecast origin, a complete 24-h horizon was generated using the seasonal-naïve, linear autoregressive, MLP, and cyclic RBF–GRNN configurations. The evaluated and selected configurations are summarised in Table 3.

The models were implemented using Python 3.12.13, NumPy 2.3.5, and scikit-learn 1.8.0. The rolling-origin protocol produces 168 out-of-sample hourly predictions and enables the models to be compared under consistent 24-h forecasting conditions. The error metrics and statistical procedures used to evaluate these predictions are defined in Section 2.5.

#### 2.4.7. Exploratory LSTM Comparator

An additional long short-term memory (LSTM) model serves as an exploratory comparator. It receives the preceding 24-hourly platform-derived load values as a sequence of scalar inputs and predicts the next hourly value. At each rolling origin, inputs and targets are standardised using only the available fitting history. For each position in the input sequence, the input, forget, candidate, and output gates are computed using (24)–(27).

(24) $$i_{t}=\sigma \left(W_{i}x_{t}+U_{i}h_{t-1}+b_{i}\right)$$

(25) $$f_{t}=\sigma \left(W_{f}x_{t}+U_{f}h_{t-1}+b_{f}\right)$$

(26) $$g_{t}=tanh\left(W_{g}x_{t}+U_{g}h_{t-1}+b_{g}\right)$$

(27) $$o_{t}=\sigma \left(W_{o}x_{t}+U_{o}h_{t-1}+b_{o}\right)$$

The cell and hidden states are updated according to (28) and (29)

(28) $$c_{t}=f_{t}⊙c_{t-1}+i_{t}⊙g_{t}$$

(29) $$h_{t}=o_{t}⊙tanh(c_{t})$$

The initial cell and hidden states are zero for each 24 h input sequence. Equation (30) maps the final hidden state to the next-hour prediction in standardised units, and (31) converts it to kilowatts using the mean and standard deviation of the fitting history available at that rolling origin.

(30) $${\hat{z}}_{n+1}=v^{T}h_{24}+b_{y}$$

(31) $${\hat{P}}_{n+1}=\mu_{o}+s_{o}{\hat{z}}_{n+1}$$

The network parameters minimise the mean squared error in (32), where M denotes the number of training sequences.

(32) $$L_{MSE}=\frac{1}{M}\sum_{n=1}^{M}{\left({\hat{z}}_{n+1}-z_{n+1}\right)}^{2}$$

The network consists of one LSTM layer, with candidate hidden sizes of 4, 8 and 16 units, followed by a linear output. Each configuration is trained for 300 epochs using full-batch Adam optimisation of mean squared error, a learning rate of 0.012, gradient-norm clipping at 1.0 and random seed 42. The mean daily MAE across the five validation origins is 0.746, 1.021 and 0.684 kW for 4, 8 and 16 units, respectively. The 16-unit configuration is refitted using the expanding available history at each of the seven held-out test origins. A 24 h forecast is generated recursively by appending each standardised prediction to the subsequent input sequence. The LSTM and MLP use the same historical load variable, 24 h lookback, and forecasting horizon. This LSTM comparison remains exploratory because its design follows inspection of the original four-model test results.

### 2.5. Forecasting Performance Metrics and Statistical Evaluation

Forecasting performance is evaluated over seven non-overlapping test days, comprising 168 hourly observations and seven 24 h forecast horizons. The four originally specified models use the same test days, and their selected hyperparameters remain fixed after validation. At each forecast origin, model fitting uses only observations preceding the forecast day [81,82,83]. The exploratory LSTM is evaluated on those same days, but its inclusion follows inspection of the original four-model test results; its comparison is therefore interpreted separately. Let *y_i_* denote the measured aggregate load and ${\hat{y}}_{i}^{\left(k\right)}$ the corresponding prediction generated by model *k*, with *i* = 1, …, *N_test_* and *N_test_* = 168. The forecasting error is defined in (33)

(33) $$e_{i}^{\left(k\right)}=y_{i}-{\hat{y}}_{i}^{\left(k\right)}$$

The primary accuracy measure was the mean absolute error (MAE), expressed in kilowatts and calculated using (34)

(34) $${MAE}^{\left(k\right)}=\frac{1}{N_{test}}\sum_{i=1}^{N_{test}}\left|e_{i}^{\left(k\right)}\right|$$

MAE provides a directly interpretable measure of the average deviation between the predicted and measured loads. To assign greater weight to the largest forecasting deviations, the root mean square error (RMSE) was calculated according to (35)

(35) $${RMSE}^{\left(k\right)}=\sqrt{\frac{1}{N_{test}}\sum_{i=1}^{N_{test}}{\left(e_{i}^{\left(k\right)}\right)}^{2}}$$

The mean absolute percentage error (MAPE) was used to express the average error relative to the magnitude of the load observed in (36).

(36) $${MAPE}^{\left(k\right)}=\frac{100}{N_{test}}\sum_{i=1}^{N_{test}}\left|\frac{e_{i}^{\left(k\right)}}{y_{i}}\right|$$

MAPE in (36) was evaluated only for observations satisfying *y_i_* > 0. This condition was met by all observations in the analysed test interval. Nevertheless, MAPE was interpreted jointly with the absolute error measures because percentage errors may assign disproportionate importance to observations characterised by comparatively low load values [81]. The ability of each model to reproduce the variability of the measured series was quantified through the out-of-sample coefficient of determination in (37)

(37) $$R^{2(k)}=1-\frac{\sum_{i=1}^{Ntest}{\left(y_{i}-{\hat{y}}_{i}^{\left(k\right)}\right)}^{2}}{\sum_{i=1}^{Ntest}{\left(y_{i}-\bar{y}\right)}^{2}},\;\bar{y}=\frac{1}{N_{test}}\sum_{i=1}^{N_{test}}y_{i}$$

Values of *R*^2^ approaching unity indicate that the forecasts reproduce a large proportion of the variability observed during the test period. Because *R*^2^ alone does not provide a direct measure of the forecasting error, it was considered together with MAE, RMSE and MAPE. The largest individual forecasting deviation was additionally determined through the maximum absolute error (38)

(38) $${MaxAE}^{\left(k\right)}=\underset{1\leq i\leq N_{\mathrm{test}}}{\max}\left|e_{i}^{\left(k\right)}\right|$$

*MaxAE* in (38) provides information on the worst hourly prediction produced by each model and is particularly relevant when occasional load peaks must be represented correctly. The forecasting improvement relative to the seasonal-naïve benchmark was quantified using the MAE skill score defined in (39)

(39) $$S_{MAE}^{\left(k\right)}=100\;\left(1-\frac{{MAE}^{\left(k\right)}}{{MAE}^{SN}}\right)$$

where ${MAE}^{SN}$ denotes the error obtained using the seasonal-naïve forecast. Positive values of $S_{MAE}^{\left(k\right)}$ indicate an improvement over the benchmark, whereas negative values indicate that the model performs less accurately than the direct repetition of the previous daily profile. The inclusion of this comparison prevents the apparent performance of the neural models from being assessed exclusively against other parameterised approaches [84,85]. In addition to the metrics calculated over the complete set of 168 observations, the daily MAE was determined separately for each test origin (40)

(40) $${MAE}_{j}^{\left(k\right)}=\frac{1}{24}\sum_{h=1}^{24}\left|y_{j,h}-{\hat{y}}_{j,h}^{\left(k\right)}\right|,\;j=1,\;\ldots ,\;7.$$

The mean and sample standard deviation of the seven daily MAE values were calculated using (41)

(41) $${\bar{MAE}}_{day}^{\left(k\right)}=\frac{1}{7}\sum_{j=1}^{7}{MAE}_{j}^{\left(k\right)},\;s_{MAE}^{\left(k\right)}=\sqrt{\frac{1}{6}\sum_{j=1}^{7}{\left({MAE}_{j}^{\left(k\right)}-{\bar{MAE}}_{day}^{\left(k\right)}\right)}^{2}}.$$

This daily analysis makes it possible to distinguish a model providing consistently accurate forecasts from one whose aggregate performance is dominated by a limited number of favourable or unfavourable days. Uncertainty was assessed through a paired block-bootstrap procedure. The complete 24 h test days were treated as indivisible temporal blocks to preserve the dependence between errors belonging to the same daily profile.

A total of *B* = 10,000 bootstrap replicates were generated by sampling seven daily blocks with replacement, using a fixed random seed of 42. For each replicate, MAE, RMSE, MAPE, and *R*^2^ were recalculated. Pairwise differences from the seasonal-naïve benchmark were determined using (42):

(42) $${∆}_{b}^{\left(k\right)}={MAE}_{b}^{\left(k\right)}-{MAE}_{b}^{\left(SN\right)},\;b=1,\;\ldots ,\;B.$$

The same resampled daily indices were applied simultaneously to each forecasting model and to the seasonal-naïve benchmark, thereby preserving the paired structure of the comparison. The 95% percentile confidence interval associated with a generic bootstrap statistic *T* was obtained as (43)

(43) $${CI}_{95\%}\left(T\right)=\left[Q_{0.025}\left({\left\{T_{b}\right\}}_{b=1}^{B}\right),\;Q_{0.975}\left({\left\{T_{b}\right\}}_{b=1}^{B}\right)\right],$$

where *Q*_0.025_ and *Q*_0.975_ denote the corresponding empirical quantiles. For the paired MAE difference in (42), negative values favour model *k*, whereas positive values favour the seasonal-naïve benchmark. A confidence interval excluding zero indicates that the observed difference remains consistent across the resampled daily profiles. Section 3 reports the aggregate metrics, the variability of the daily errors, the improvement relative to the seasonal-naïve benchmark and the associated block-bootstrap confidence intervals.

Consequently, every evaluation criterion introduced in this subsection is accompanied by a corresponding quantitative result.

## 3. Results

This section reports the physical implementation of the monitoring architecture, verifies the quality and statistical properties of the exported dataset, documents hyperparameter selection over the rolling-validation interval and compares the forecasting models over seven out-of-sample test days. The organisation mirrors the methodological sequence introduced in Section 2, thereby providing graphical or quantitative evidence for each stage of the proposed workflow.

### 3.1. Hardware Implementation and Field Deployment

The electronic interface designed using EasyEDA was manufactured, populated, and integrated with the Arduino Uno acquisition unit and the Raspberry Pi single-board computer. The realised PCB provides dedicated screw-terminal connections for the SCT current-sensor channels and the electronic interface required to convey the conditioned signals to the acquisition stage. The Arduino Uno performs local signal acquisition, whereas the Raspberry Pi manages buffering, storage, and communication with the remote platform. Communication between the two digital units is established through a USB connection. A local acquisition and communication assembly was installed at each of the 14 wastewater pumping stations. The sensing configuration of each station was adapted to the number of installed pumps. Each of the 23 three-phase pumps was associated with three current-sensing channels, resulting in 69 channels across the complete distributed installation. The clamp-type sensors allowed the electrical variables to be acquired without introducing series measurement components into the pump power conductors. Figure 5 documents the realised monitoring assembly and its integration into the electrical control cabinet.

The installation demonstrates that the sensing, acquisition, local processing and communication functions were integrated within the existing cabinet rather than evaluated as disconnected laboratory components. The realised hardware consequently provides the physical implementation of the conceptual architecture described in Section 2.1 and the measurement configuration specified in Section 2.2.

### 3.2. Dataset Characterisation and Quality Assessment

This completeness check applies only to the surviving hourly export. It cannot verify the original minute-level acquisitions, the current-to-load conversion, or the hourly aggregation performed by the legacy platform. The observation period also excludes any assessment of seasonal variability and does not establish performance during rainfall-driven inflows or different dry-weather operating regimes. The missing-data rate defined in (10) is therefore 0%. The hourly export contains no null, negative, or zero load values. Each sequential day contained exactly 24 ordered hourly positions; the hour variable progressed from 1 to 24, and the day-of-week variable followed its expected seven-day cyclic sequence. No duplicated hourly positions were detected within any sequential day. The chronological behaviour and empirical distribution of the aggregate load are presented in Figure 6. Figure 6a also identifies the three intervals used in the forecasting analysis: 432 observations for initial training, 120 observations corresponding to five rolling-validation days, and 168 observations corresponding to seven rolling-test days.

The complete series ranged from 9.326 to 16.012 kW. The mean load was 13.128 kW, while the median was 13.595 kW. The difference between these values is consistent with the recurrent low-load intervals visible in Figure 6a and with the lower-load portion of the distribution in Figure 6b. The sample standard deviation was 1.816 kW, and the coefficient of variation was 13.83%, indicating appreciable temporal variability in the aggregate electrical demand. The descriptive statistics and quality indicators are summarised in Table 4.

Table 4 reports the dataset characteristics and quality-verification outcomes together with descriptive statistics of the aggregate load. The results confirm that all 720 expected observations were available and chronologically consistent. Therefore, the dataset was retained without interpolation, replacement of extreme observations, or removal of load peaks, and its original temporal ordering was preserved throughout the forecasting analysis. The values reported in Table 4 quantitatively verify the completeness and variability checks defined in (8)–(10).

### 3.3. Hyperparameter Selection

Hyperparameters are selected using the five rolling-validation origins defined in Section 2.4. At each origin, a 24 h forecast was generated, and the mean daily MAE was calculated. Selection was conducted independently within each model family, without using any observation belonging to the final seven test days. The validation results are shown in Figure 7.

For the linear autoregressive model, the validation MAE generally decreased as the delay increased. The minimum value of 0.666 kW was obtained with *d* = 96. A delay of 48 h produced a slightly higher MAE of 0.678 kW, whereas delays shorter than 24 h resulted in markedly larger errors. The selected configuration therefore used the previous 96 observations. The MLP results in Figure 7b show that performance depended jointly on the delay and the hidden-layer size. The minimum validation MAE was 0.503 kW for d = 24 and H = 16. Increasing the hidden layer to 32 neurons with the same delay produced a similar but slightly higher MAE of 0.510 kW. The selected configuration therefore combined one complete daily load cycle with 16 hidden neurons, avoiding additional model complexity that did not improve validation accuracy. For the cyclic RBF–GRNN, the lowest validation MAE was 0.543 kW at σ = 0.30. The error remained relatively stable between approximately σ = 0.20 and 0.35, after which it increased progressively as the Gaussian functions became wider. This behaviour indicates that local calendar-based responses were more effective than extensive smoothing for representing the observed day–hour relationship.

The configurations in Table 5 remain fixed before testing in the original four-model analysis. At each test origin, the three fitted models use the expanding history available before that forecast day; their selected delay, hidden-layer size, and Gaussian spread remain fixed. The seven test days do not enter their hyperparameter selection. The exploratory LSTM follows the same origin-wise fitting rule, but its inclusion during manuscript revision follows inspection of the original test results.

### 3.4. Rolling-Origin Forecasting Performance and Uncertainty

Figure 8a compares the measured load with the forecasts generated over the seven rolling-test days. The test observations ranged from 9.609 to 15.901 kW and retained a recurrent intraday pattern characterised by lower early-hour values followed by transitions towards higher-load intervals. All four models reproduced the principal daily cycle, although their ability to represent changes in amplitude differed across the seven forecast origins.

The seasonal-naïve benchmark reproduced the test profile effectively when consecutive days exhibited similar amplitudes. Its largest deterioration occurred on the fifth test day, for which the daily MAE increased to 1.069 kW. The linear autoregressive model produced comparatively large errors on the first and seventh days and tended to smooth some of the high-load variations. The MLP provided more uniform performance, with daily MAE values ranging from 0.286 to 0.575 kW. The RBF–GRNN ranged from 0.242 to 0.701 kW, providing the lowest daily error on the fifth test day but a higher error on the second day. The aggregate forecasting metrics and their block-bootstrap confidence intervals are reported in Table 6.

Among the four originally specified models, the MLP has the lowest MAE (0.441 kW) and MAPE (3.266%), corresponding to improvements of 12.41% and 15.29%, respectively, relative to the seasonal-naïve benchmark. Its daily MAE was 0.441 ± 0.104 kW, compared with 0.504 ± 0.257 kW for the benchmark, 0.535 ± 0.225 kW for the linear autoregressive model, and 0.451 ± 0.152 kW for the RBF–GRNN. Within the seven observed test days, the MLP has the lowest average absolute error among those four models. This descriptive ranking does not establish superiority over the seasonal-naïve benchmark. The RBF–GRNN achieved the lowest RMSE (0.525 kW), maximum absolute error (1.214 kW), and highest out-of-sample *R*^2^ (0.918). It therefore showed a complementary advantage in limiting larger forecasting deviations and reproducing the variability of the exported aggregate-load series. The paired MAE differences relative to the seasonal-naïve benchmark were +0.031 kW for the linear model, −0.063 kW for the MLP and −0.053 kW for the RBF–GRNN. Their corresponding 95% confidence intervals were [−0.161, 0.215], [−0.246, 0.057] and [−0.339, 0.143] kW. Because all intervals included zero, the available test data do not establish a statistically conclusive difference from the benchmark. The numerical results should therefore be interpreted as evidence of complementary empirical behaviour rather than universal superiority of a single model. The exploratory LSTM has a test MAE of 0.424 kW, compared with 0.441 kW for the MLP and 0.504 kW for the seasonal-naïve benchmark. However, its RMSE (0.637 kW) and maximum absolute error (2.959 kW) were higher than those of the MLP (0.568 and 1.369 kW, respectively). The standard deviation of the LSTM’s seven daily MAE values was 0.298 kW, compared with 0.104 kW for the MLP. The paired mean daily MAE difference, calculated as LSTM minus MLP, was −0.017 kW, with a 95% daily-block bootstrap confidence interval of [−0.225, 0.262] kW. The corresponding difference relative to the seasonal-naïve benchmark was −0.080 kW, with a confidence interval of [−0.402, 0.249] kW. Both intervals included zero. The slightly lower aggregate MAE of this exploratory LSTM therefore does not establish an advantage over the MLP or the seasonal-naïve model, and its larger peak error warrants caution.

## 4. Discussion

The rolling-origin evaluation shows that forecasting performance depends on both temporal representation and multi-step generation strategy. The seasonal-naïve model constituted a strong reference because the aggregate-load series exhibits a recurrent 24 h structure. The linear autoregressive model did not improve average forecasting accuracy despite using 96 delayed observations, indicating that a longer linear history was insufficient to represent changes in daily amplitude. Among the four originally specified models, the MLP, using the preceding 24 h and 16 hidden neurons, has the lowest MAE and MAPE. Conversely, the cyclic RBF–GRNN achieved the lowest RMSE and maximum absolute error and the highest *R*^2^. These complementary outcomes are consistent with evidence that no forecasting architecture is uniformly optimal across aggregation levels, temporal horizons, and input representations [47,78]. The exploratory LSTM attained a slightly lower aggregate MAE than the MLP, but a higher RMSE and maximum absolute error; the paired confidence interval for their MAE difference included zero. Its numerical ranking cannot be interpreted as evidence that recurrent modelling is superior on this 30-day series. The paired block-bootstrap analysis prevents the numerical ranking from being overstated. The confidence intervals of all MAE differences relative to the seasonal-naïve benchmark included zero. Accordingly, the seven held-out daily forecast origins support differences in empirical error behaviour but do not provide statistically conclusive evidence of superiority. The 10,000 bootstrap replications resample only seven distinct daily test blocks and do not increase the number of independently observed operating days. The resulting intervals should therefore be interpreted cautiously, particularly when assessing whether the observed rankings would persist under different operating or seasonal conditions. The MLP was more consistent in terms of average absolute error, whereas the RBF–GRNN was more effective in limiting larger deviations. Selection between these configurations would therefore depend on whether the operational priority is average accuracy, containment of peak errors, interpretability, or computational demand. The principal novelty of the study lies in the end-to-end integration of distributed current sensing, custom electronic interfacing, Arduino Uno acquisition, Raspberry Pi-based storage and communication, platform-level aggregation, and infrastructure-scale forecasting. IoT electricity meters commonly integrate measurement and remote data access [5], whereas pumping-station studies frequently focus on equipment-level parameter prediction or alarm generation [7]. The present work instead connects 69 sensing channels distributed across 14 stations to a common aggregate forecasting target. Equipment-level diagnosis and aggregate-load forecasting should be considered complementary functions: the former supports maintenance, while the latter can support demand assessment, peak identification, and future coordinated scheduling. Recent studies have modelled energy consumption in wastewater pumping systems using deep learning [4] and investigated simulation-based optimisation of pumping operations [42]. These studies provide application-specific context, but their objectives, datasets and evaluation targets differ from the present 24-h forecasting of a platform-derived aggregate-load series. Their published numerical outcomes therefore cannot be treated as a direct performance benchmark for the models evaluated here. The scope of the electrical quantity must nevertheless be interpreted carefully. The archived dataset contains the aggregate-load estimate exported by the legacy platform rather than raw voltage and current waveforms. Active power cannot be independently reconstructed from current observations alone without simultaneous voltage, phase-displacement, and power-factor information. The reported kilowatt values must therefore be interpreted as platform-derived load estimates. Moreover, the surviving documentation does not include complete channel-by-channel calibration records or a traceable uncertainty budget. A future metrological campaign should compare the sensing channels with calibrated reference instrumentation and quantify linearity, repeatability, inter-channel variability, conversion uncertainty and communication reliability. Such a campaign should document the burden-resistor and analogue-component values, conditioning-circuit topology, ADC reference voltage, resolution, sampling strategy and RMS-estimation procedure. It should also acquire simultaneous voltage and current waveforms and compare each measurement channel with a calibrated three-phase power analyser over representative current amplitudes, pump operating conditions, waveform distortions and power factors. Until those measurements are available, the results establish forecasting performance for the archived platform variable, not the metrological accuracy of active-power measurement. The evaluated models also present different implementation trade-offs. The linear autoregressive model has low computational and memory requirements but limited nonlinear representation. The MLP remains compact, although recursive generation of a 24 h horizon can propagate preceding prediction errors. The RBF–GRNN produces the daily profile directly from cyclic calendar variables, but its memory and computational requirements increase with the number of fitting samples. These aspects should be quantified before inference is transferred to the Raspberry Pi or another edge platform. Cross-domain sensor-system studies similarly indicate that the transducer, electronic interface, and signal-processing strategy should be evaluated as coordinated elements of the same architecture [79]. Finally, the 30-day dataset and seven test origins limit seasonal and operational generalisation. The available observations cannot establish robustness under long-term changes in demand, exceptional inflow conditions, altered pump utilisation or maintenance events. Furthermore, the models use only historical aggregate load or calendar variables; reservoir level, flow, pump state, weather, and additional electrical quantities were unavailable. Consequently, this study cannot determine whether adding wet-well level, inflow, rainfall or pump-state measurements would improve forecasting performance. Nor does it demonstrate energy savings or improved pump scheduling resulting from the forecasts; these remain objectives for subsequent operational evaluations. Longer multi-season monitoring campaigns should therefore combine traceable electrical measurements with operational covariates, probabilistic forecasting and explicit evaluations of computational latency, memory, energy consumption and communication reliability. Within its defined scope, the study demonstrates the feasibility of an integrated sensing-to-forecasting workflow rather than the universal superiority of a forecasting model or the metrological performance of a power analyser.

## 5. Conclusions

This study presents the field deployment of a distributed non-invasive electrical monitoring architecture combining SCT current sensors, custom interfaces designed in EasyEDA, Arduino Uno-based data acquisition, Raspberry Pi-based storage and communication, and a remote aggregation platform. The installation covers 14 wastewater pumping stations, 23 three-phase pumps, and 69 sensing channels. The resulting dataset comprised 720 consecutive hourly platform-derived aggregate-load estimates. Forecasting performance was evaluated through five rolling-validation origins and seven held-out test origins. Among the four originally specified models, the MLP has the lowest MAE (0.441 kW) and MAPE (3.266%), whereas the RBF–GRNN achieved the lowest RMSE (0.525 kW), maximum absolute error (1.214 kW) and highest R^2^ (0.918). Because the paired bootstrap confidence intervals for the MAE differences relative to the seasonal-naïve benchmark included zero, the observed improvements cannot be considered statistically conclusive. An exploratory LSTM comparison yielded a slightly lower MAE (0.424 kW) than the MLP, but larger RMSE and maximum absolute error; the paired MAE difference between the networks was also inconclusive. The results instead demonstrate complementary model behaviour under a common leakage-free evaluation protocol. The contribution of the study lies in documenting a field-deployed sensing-to-forecasting workflow and in evaluating forecasting methods on the surviving platform-derived aggregate-load export. The study does not validate the metrological accuracy of the original sensing chain or demonstrate that the fitted forecasting models outperform the seasonal-naïve benchmark. Its present validity is limited to the platform-derived variable and the analysed monitoring interval. Future work should perform traceable metrological validation, extend acquisition across multiple seasons, incorporate operational and environmental variables, and quantify the computational requirements associated with edge-based forecasting.

## Figures and Tables

**Figure 1:**
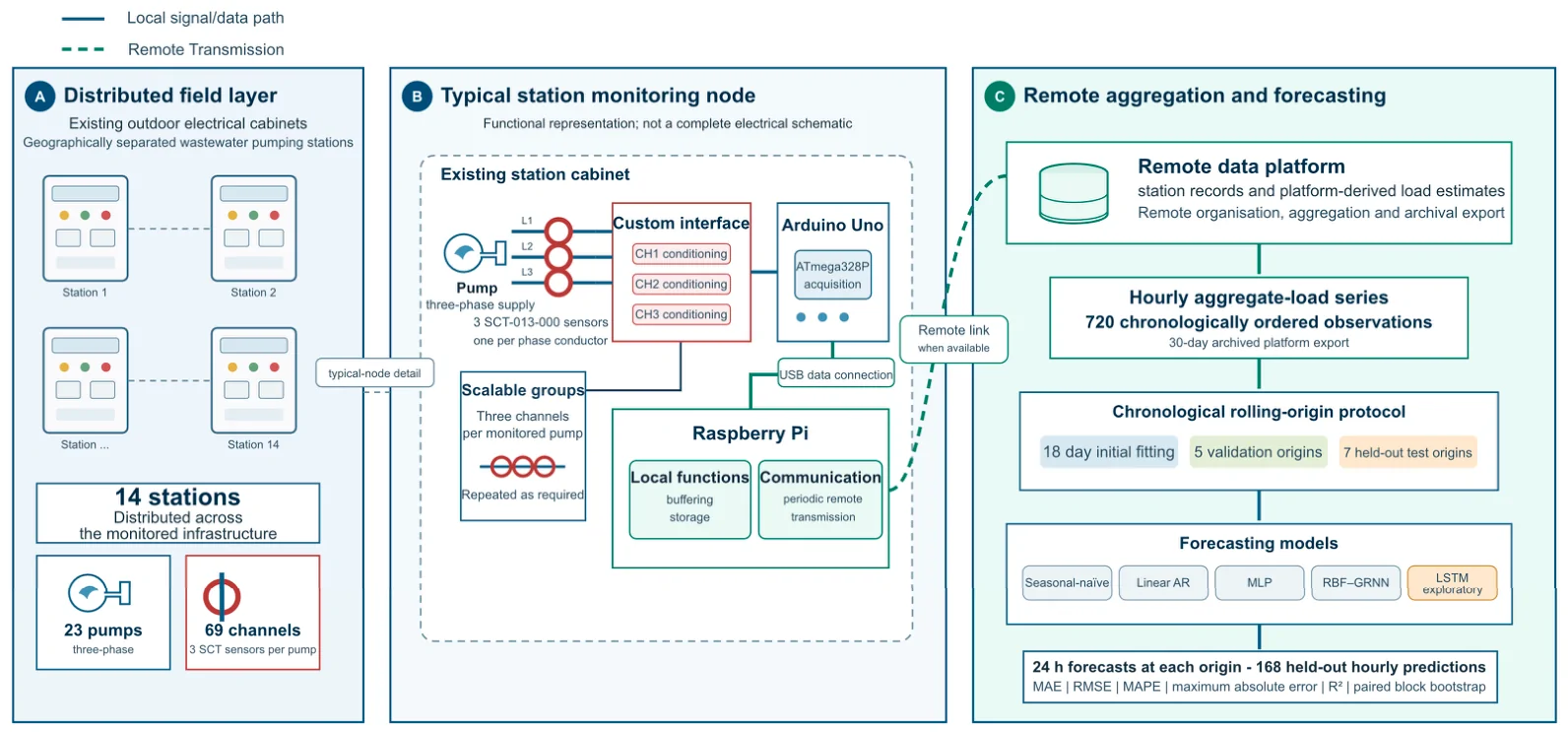
End-to-end architecture of the distributed monitoring and forecasting system: (**A**) field deployment; (**B**) typical station monitoring node; and (**C**) remote data aggregation and chronological 24 h load forecasting. Solid lines indicate local signal and data paths, whereas the dashed line indicates remote transmission.

**Figure 2:**
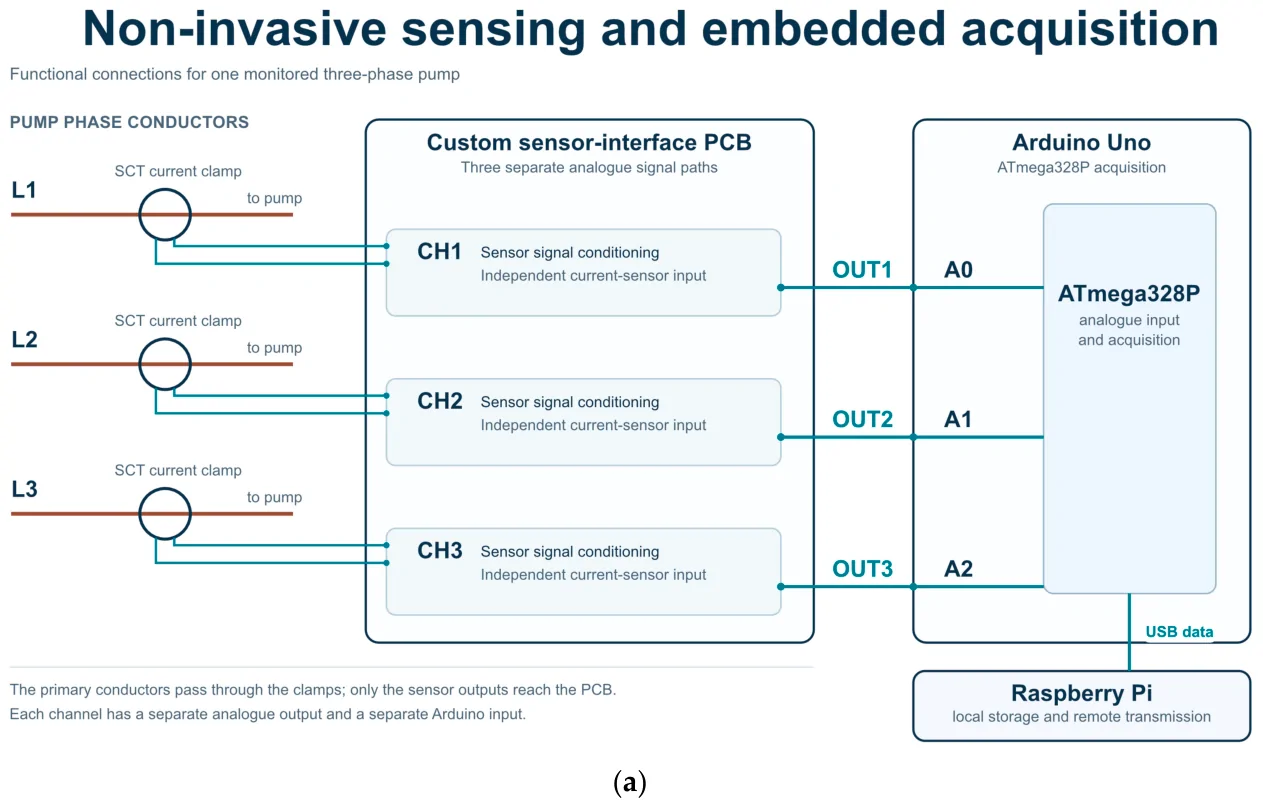

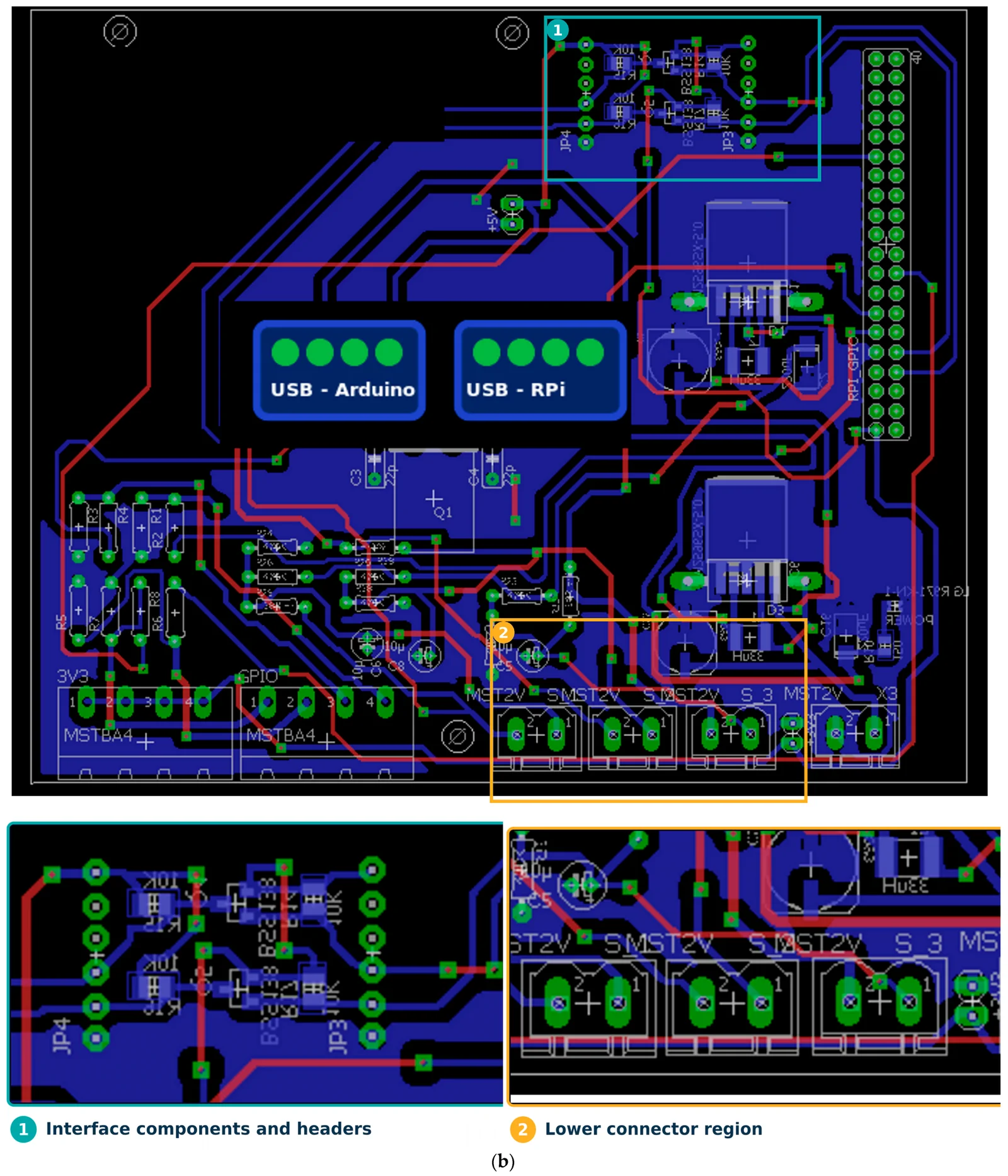
Available electronic documentation of the monitoring interface: (**a**) functional representation of one three-phase sensing and acquisition group; (**b**) PCB layout developed using EasyEDA. The available documentation does not constitute a complete metrological circuit description.

**Figure 3:**
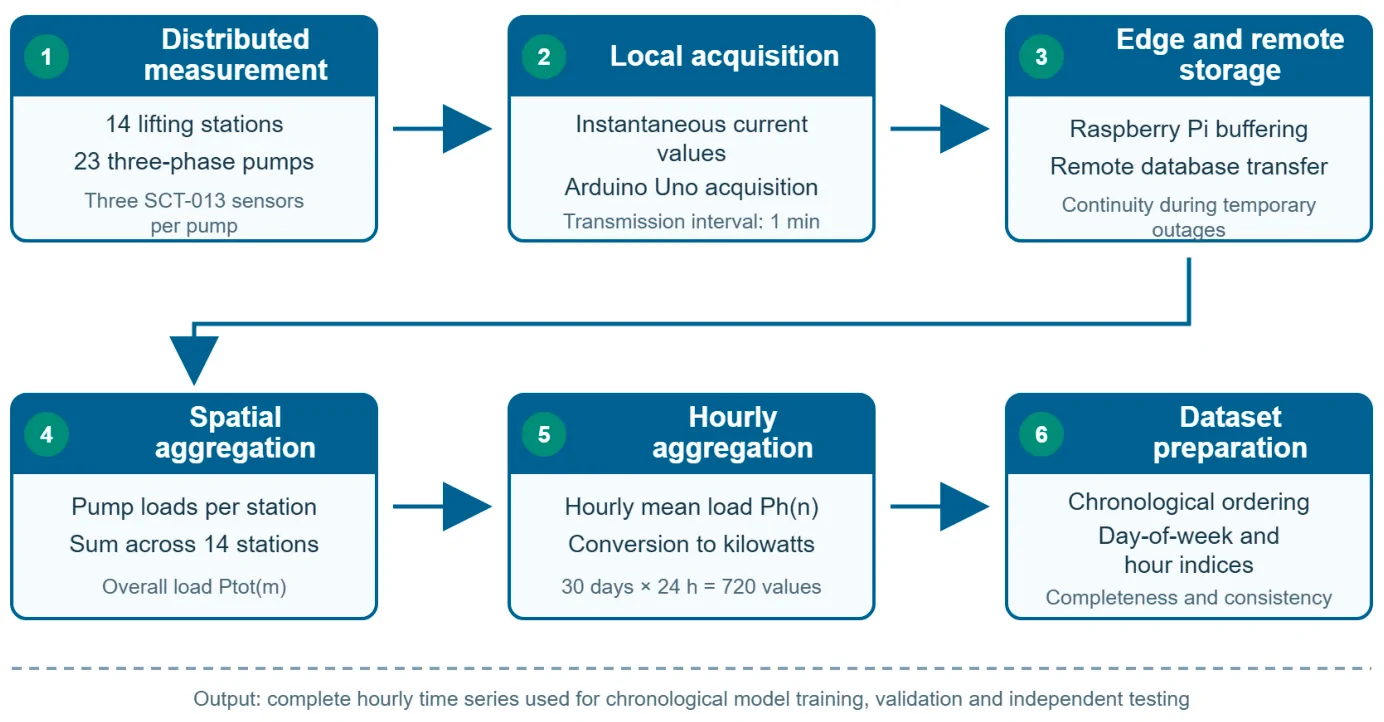
Acquisition and pre-processing workflow used to construct the hourly aggregate-load series.

**Figure 4:**
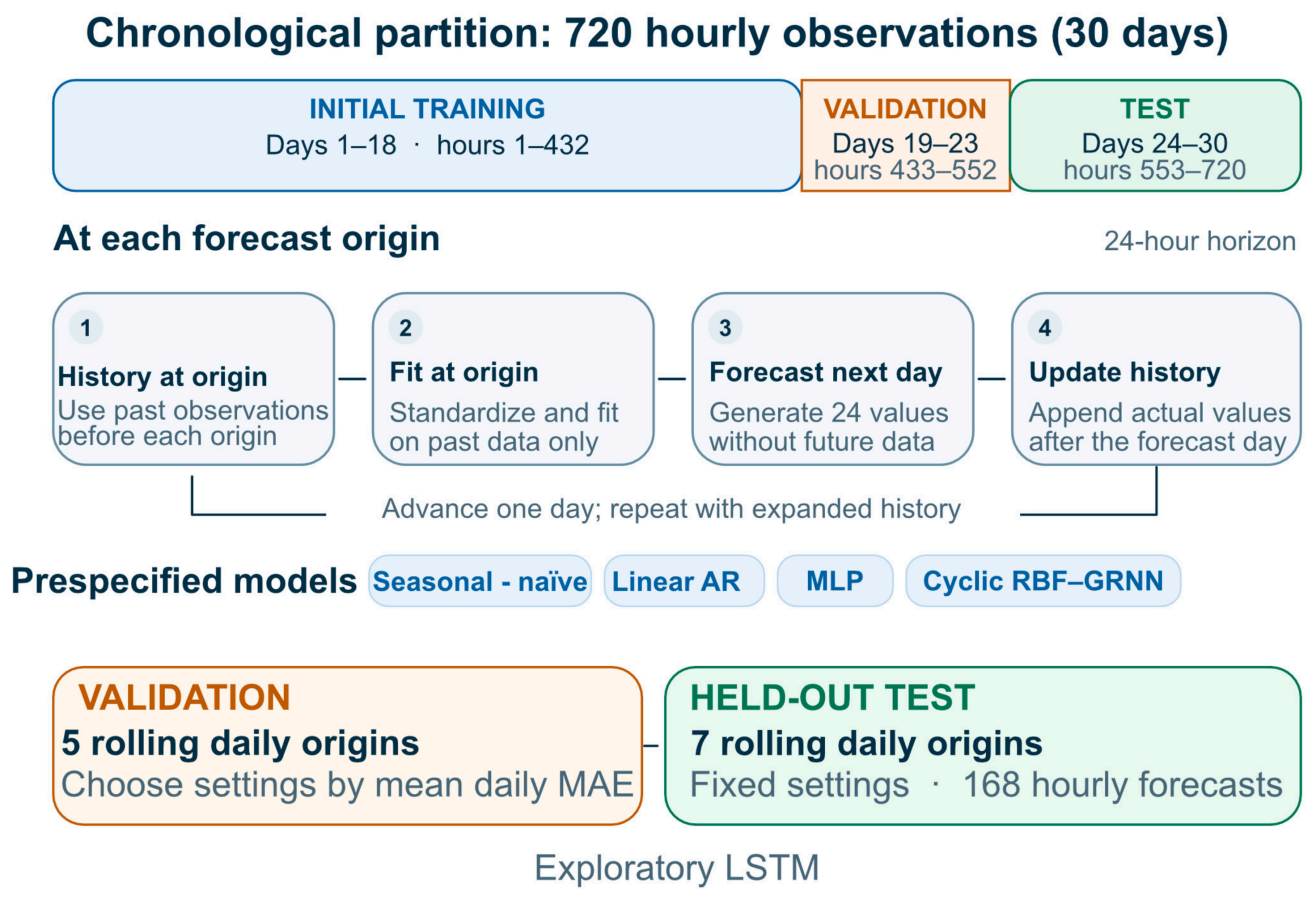
Rolling-origin procedure used for model selection and seven-day out-of-sample evaluation.

**Figure 5:**
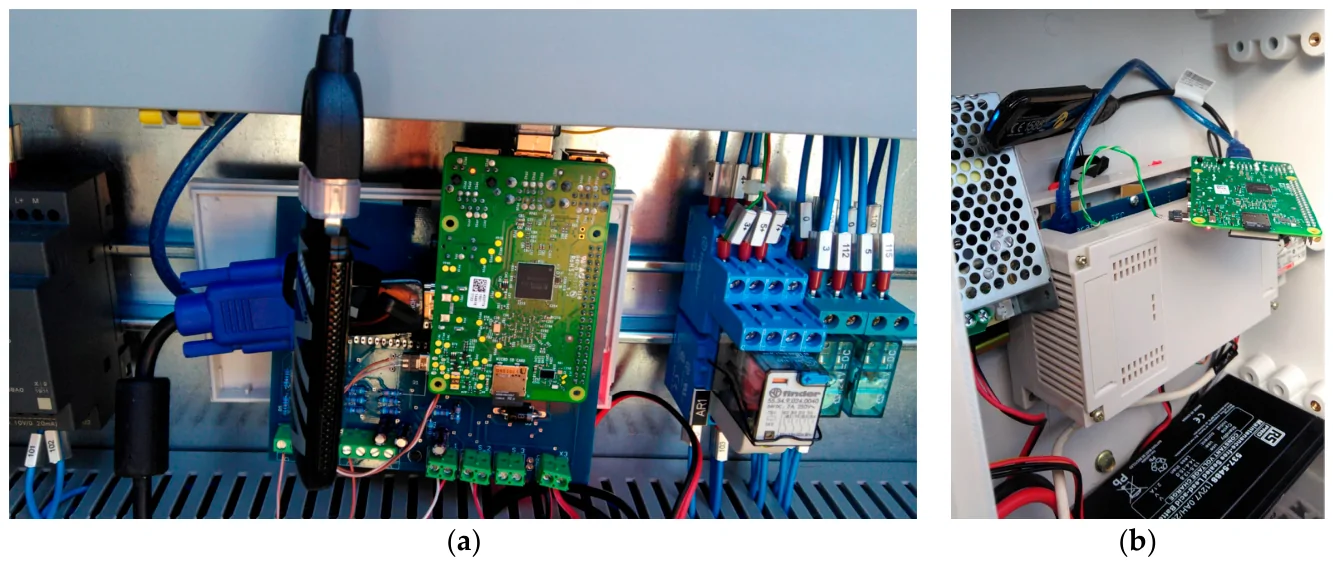
Realised monitoring node installed in the pumping-station control cabinet: (**a**) front view of the custom interface and local processing unit; (**b**) side view showing the processing, communication and power-supply components.

**Figure 6:**
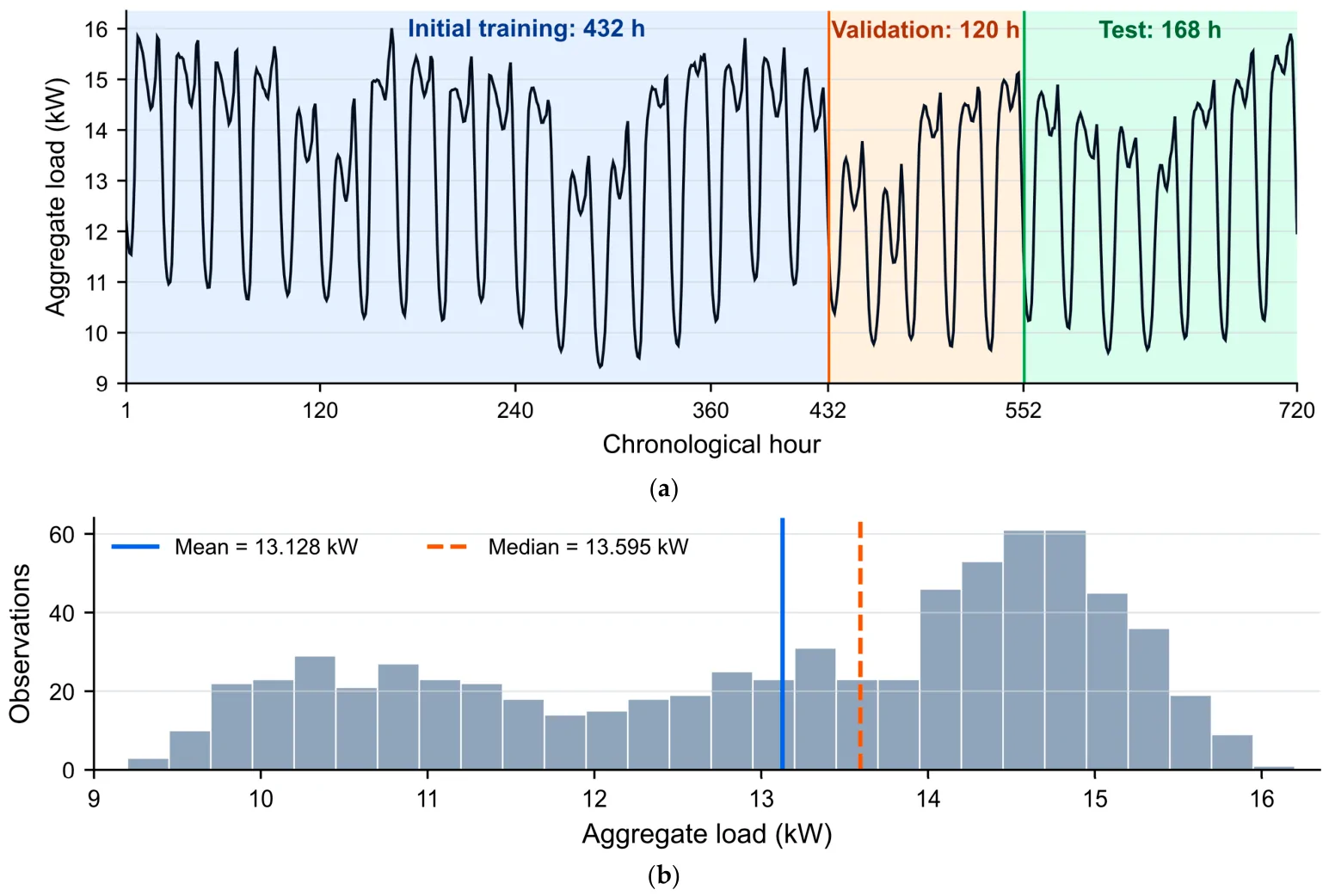
Platform-derived aggregate load: (**a**) chronological series with initial-training, validation, and test partitions; (**b**) empirical distribution with mean and median values.

**Figure 7:**
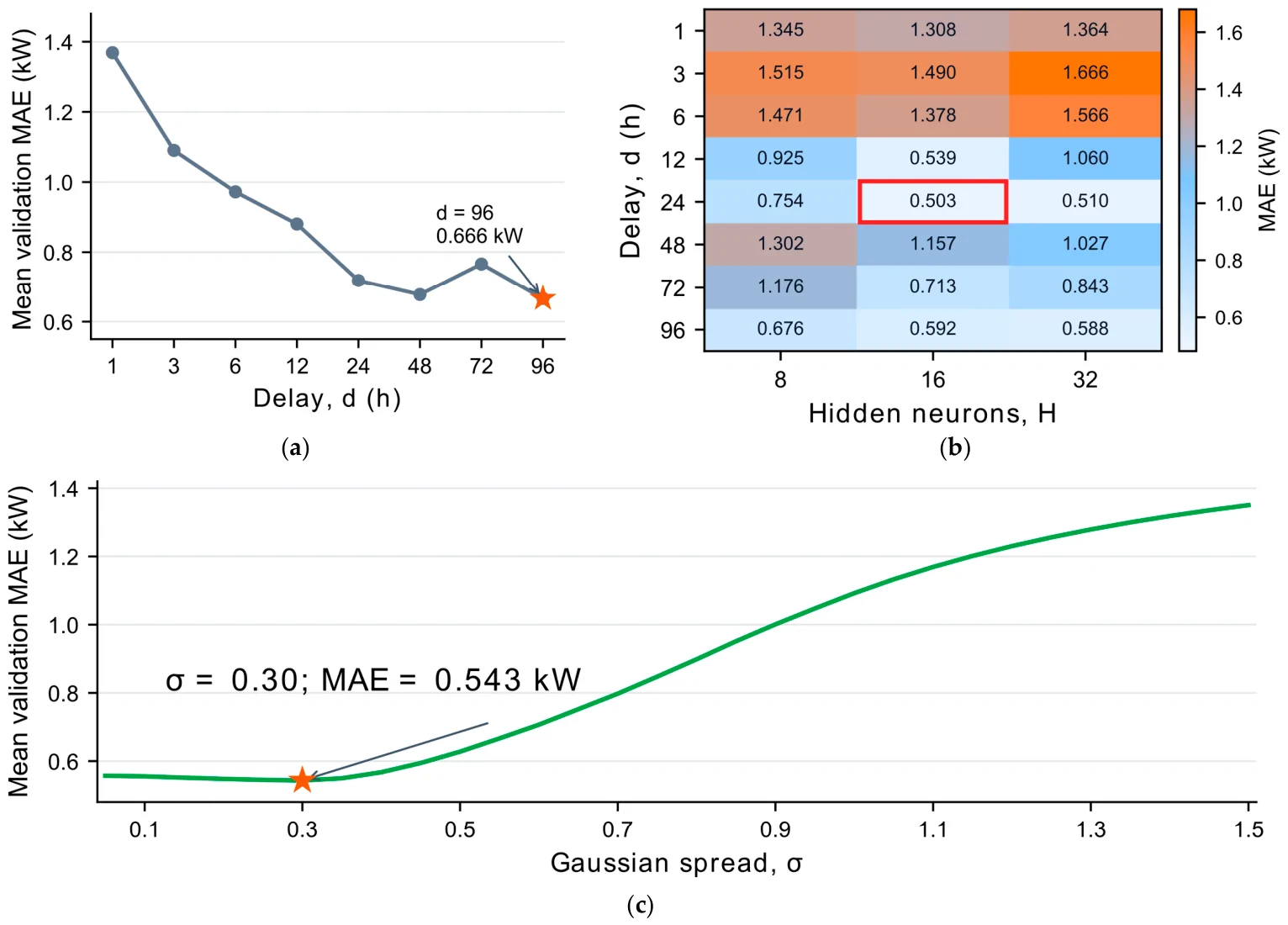
Mean daily validation MAE across five rolling origins: (**a**) linear autoregressive delay; (**b**) MLP delay–hidden-unit combinations; (**c**) RBF–GRNN Gaussian spread. The highlighted configurations were selected before testing; the red box in panel (**b**) identifies the selected MLP configuration (d = 24 and H = 16).

**Figure 8:**
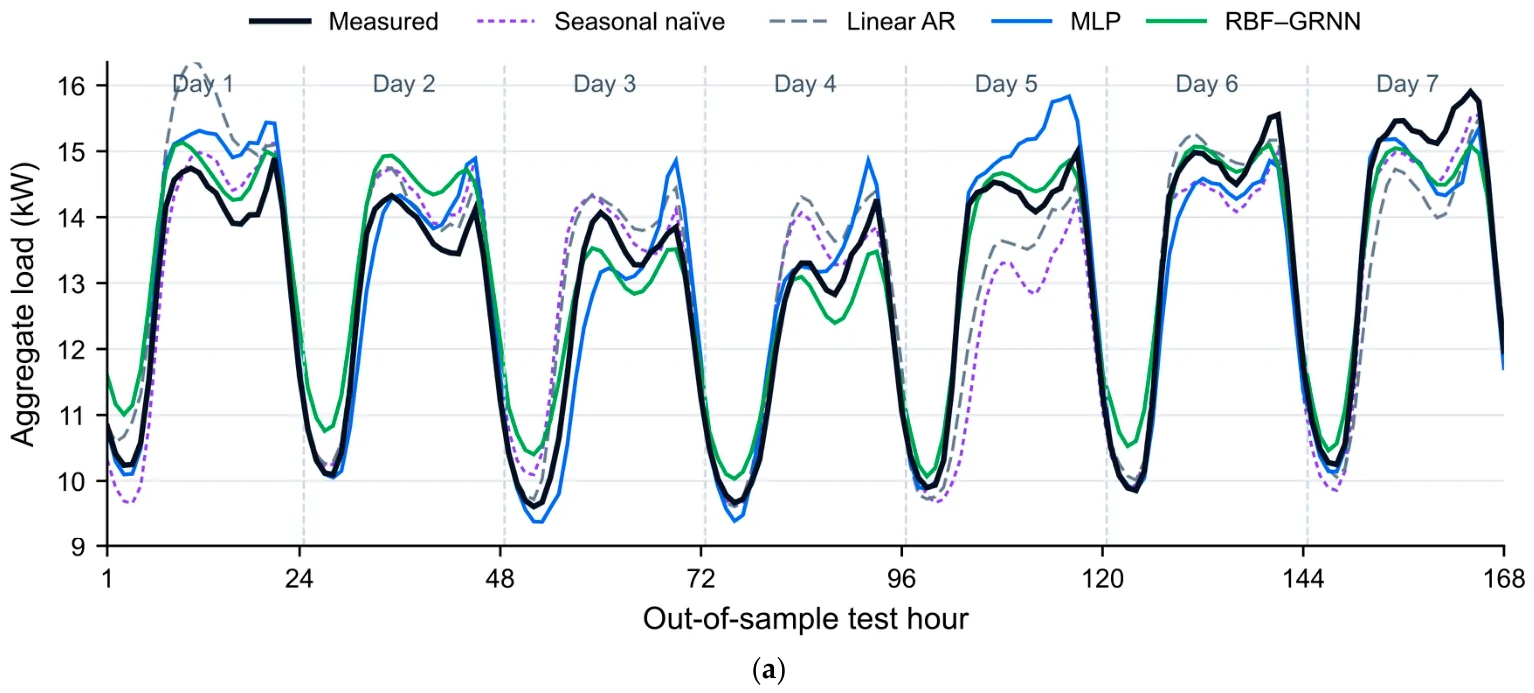

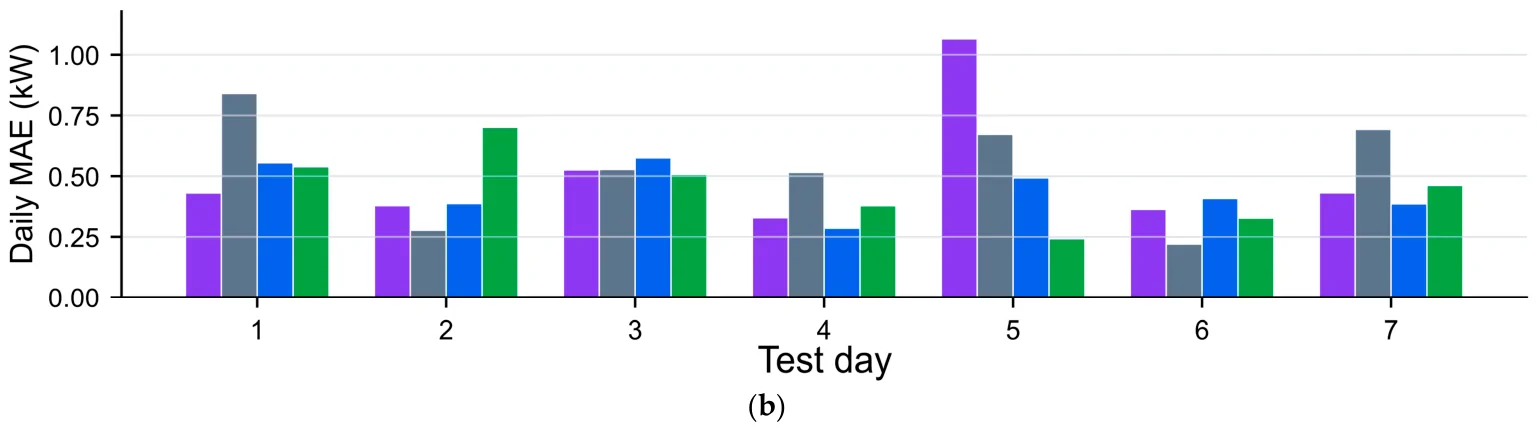
Seven-day out-of-sample evaluation of the four prespecified models: (**a**) measured and forecast platform-derived aggregate load over 168 h; (**b**) daily MAE. The exploratory post hoc LSTM is excluded from this figure and is reported separately in Table 6.

**Table 1:** Field configuration and measurement strategy.

Parameter	Design Value or Available Information	Function or Limitation
Sensor technology	SCT-013-000 split-core AC current transformer	Non-invasive phase-current sensing
Measured electrical quantity	Phase current only	No voltage waveform or power-factor measurement
Nominal sensor sensitivity	0.5 mA/A	Manufacturer-specified transformation ratio
Manufacturer specifications	Accuracy ±1%; linearity ±3%	Component specifications, not system-level validation
Exported-data resolution	1 h	Temporal resolution used for forecasting
Monitoring sequence	30 complete days	Chronological observation period
Aggregate observations	720	Input time series for forecasting
Forecasting target	Hourly platform-derived aggregate-load estimate (kW)	Combined load estimate for the 14 stations

**Table 2:** Structure of the time series used for forecasting the total load.

Variable	Symbol or Value	Role
Phase-current observation	*I_s,p,l_*(*m*)	Current value acquired from phase *l* of pump *p*
Platform-derived pump load	$P_{s,p}^{plat}\left(m\right)$	Load estimate associated with an individual pump
Station load	*P_s_*(*m*)	Sum of the loads of the pumps installed at station *s*
Infrastructure load	*P_tot_*(*m*)	Combined load of the 14 stations
Hourly forecasting variable	*P_kW_*(*n*)	Aggregate-load target expressed in kW
Sequential day	*q* = 1, …, 30	Chronological position within the observation period
Day of the week	*d*(*n*) = 1, …, 7	Weekly calendar information
Hour of the day	*h*(*n*) = 1, …, 24	Daily calendar information
Temporal resolution	∆*t* = 1 h	Interval between consecutive forecasting samples
Number of observations	*N* = 720	Complete hourly time series

**Table 3:** Configurations of the four originally specified forecasting models selected through rolling-origin validation.

Model	Input Variables	Candidate Configurations	Selected Configuration	24 h Forecast Mode
Seasonal-naïve	Previous-day load profile	No fitted hyperparameters	Seasonal delay of 24 h	Direct
Linear autoregressive	Standardised lagged loads	d ∈ {1,3,6,12,24,48,72,96}	D = 96	Recursive
MLP	Standardised lagged loads	d ∈ {1, 3, 6, 12, 24, 48, 72, 96}; H ∈ {8, 16, 32}	*d* = 24; *H* = 16	Recursive
RBF–GRNN	Cyclic hour and day-of-week variables	σ ∈ {0.05, 0.10, …, 1.50}; step = 0.05	Σ = 0.30	Direct

**Table 4:** Dataset structure, quality-verification outcomes, and descriptive statistics of the aggregate load.

Dataset Characteristic or Quality Indicator	Verification Outcome	Load-Distribution Statistic	Computed Value
Number of observations, *N*	720	Mean	13.128 kW
Monitoring duration	30 days	Sample standard deviation	1.816 kW
Temporal resolution	1 h	Median	13.595 kW
Missing observations	0	Minimum	9.326 kW
Missing-data rate	0%	First quartile, Q1	11.445 kW
Temporal duplicates	0	Third quartile, Q3	14.660 kW
Negative or zero load values	0	Maximum	16.012 kW
Incomplete daily sequences	0	Coefficient of variation	13.83%

**Table 5:** Hyperparameters of the three originally specified fitted models selected using five rolling-validation origins.

Model	Selected Configuration	Mean Validation MAE (kW)
Linear autoregressive model	*d* = 96	0.666
Backpropagation MLP	*d* = 24; *H* = 16	0.503
Cyclic RBF–GRNN	σ = 0.30	0.543

**Table 6:** Forecasting performance across seven held-out daily origins. Values in brackets are 95% confidence intervals obtained from 10,000 bootstrap resamplings of complete 24 h blocks.

Model	MAE (kW), 95% CI	RMSE (kW), 95% CI	MAPE (%), 95% CI	R^2^, 95% CI	MaxAE (kW)	MAE Skill Score (%)
Seasonal-naïve	0.504 [0.375, 0.704]	0.693 [0.435, 0.965]	3.856 [2.828, 5.299]	0.857 [0.705, 0.947]	3.083	0.00
Linear AR	0.535 [0.380, 0.684]	0.701 [0.507, 0.855]	4.013 [2.892, 5.059]	0.854 [0.768, 0.924]	2.096	−6.18
MLP	0.441 [0.370, 0.513]	0.568 [0.486, 0.638]	3.266 [2.750, 3.839]	0.904 [0.863, 0.935]	1.369	12.41
RBF–GRNN	0.451 [0.349, 0.555]	0.525 [0.422, 0.616]	3.641 [2.815, 4.511]	0.918 [0.866, 0.950]	1.214	10.56
LSTM	0.424 [0.259, 0.652]	0.637 [0.356, 0.919]	3.312 [1.997, 5.248]	0.880 [0.727, 0.962]	2.959	15.86

Note: The seasonal-naïve, linear autoregressive, MLP and RBF–GRNN models constitute the prespecified comparison. The LSTM was introduced as an exploratory post hoc comparator after inspection of the original four-model results. Its confidence intervals were calculated using the same daily block-bootstrap procedure for descriptive consistency, but its results are not included in the primary inferential comparison.

## Data Availability

The processed hourly aggregate-load dataset, chronological partitions, and rolling-origin forecasts supporting the findings of this study are provided as Supplementary Data S1. These materials permit independent verification of the reported forecasting metrics and chronological data partitions, but they do not permit reconstruction or validation of the original sensing, current-to-load conversion, or minute-to-hour aggregation procedures. The original minute-level phase-current records and the complete legacy acquisition platform software are no longer available because the original storage medium was lost.

## Supplementary Materials

The following supporting information can be downloaded at: https://www.mdpi.com/article/10.3390/s26206445/s1, Data S1: Hourly Aggregate Electrical-Load Dataset, including platform-derived hourly observations, chronological data partitions, and rolling-origin forecasts.
